# Accuracy in detecting inadequate research reporting by early career peer reviewers using an online CONSORT-based peer-review tool (COBPeer) versus the usual peer-review process: a cross-sectional diagnostic study

**DOI:** 10.1186/s12916-019-1436-0

**Published:** 2019-11-19

**Authors:** Anthony Chauvin, Philippe Ravaud, David Moher, David Schriger, Sally Hopewell, Daniel Shanahan, Sabina Alam, Gabriel Baron, Jean-Philippe Regnaux, Perrine Crequit, Valeria Martinez, Carolina Riveros, Laurence Le Cleach, Alessandro Recchioni, Douglas G. Altman, Isabelle Boutron

**Affiliations:** 1 Université de Paris, CRESS, Inserm, INRA, F75004 Paris, France; 2 Centre d’Épidémiologie Clinique, Hôpital Hôtel Dieu, Assistance Publique des Hôpitaux de Paris, Paris, France; 3 Emergency department, Service d’Accueil des Urgences, Hôpital Lariboisière, Assistance Publique des Hôpitaux de Paris, University Diderot, 75010 Paris, France; 4 Centre for Journalology, Ottawa Hospital Research Institute, School of Epidemiology and Public Health, University of Ottawa, Ottawa, Canada; 5 Department of Emergency Medicine, School of Medicine, University of California, Los Angeles, CA USA; 6 Centre for Statistics in Medicine, University of Oxford, Oxford, UK; 7 Cochrane Central Executive, London, UK; 8 Taylor and Francis group, Abingdon, UK; 9 Department of Anesthesiology, Assistance Publique Hôpitaux de Paris, Hôpital Raymond Poincaré, Garches, France; 10 Service de Dermatologie, Hopital Mondor, Assistance Publique des Hôpitaux de Paris, Université Paris Est Creteil, EpidermE, 94000 Créteil, France; 11 BMC Medicine, London, UK

**Keywords:** Peer reviewers, Randomized controlled trials, Reporting, CONSORT statement

## Abstract

**Background:**

The peer review process has been questioned as it may fail to allow the publication of high-quality articles. This study aimed to evaluate the accuracy in identifying inadequate reporting in RCT reports by early career researchers (ECRs) using an online CONSORT-based peer-review tool (COBPeer) versus the usual peer-review process.

**Methods:**

We performed a cross-sectional diagnostic study of 119 manuscripts, from BMC series medical journals, *BMJ*, *BMJ Open*, and *Annals of Emergency Medicine* reporting the results of two-arm parallel-group RCTs. One hundred and nineteen ECRs who had never reviewed an RCT manuscript were recruited from December 2017 to January 2018. Each ECR assessed one manuscript. To assess accuracy in identifying inadequate reporting, we used two tests: (1) ECRs assessing a manuscript using the COBPeer tool (after completing an online training module) and (2) the usual peer-review process. The reference standard was the assessment of the manuscript by two systematic reviewers. Inadequate reporting was defined as incomplete reporting or a switch in primary outcome and considered nine domains: the eight most important CONSORT domains and a switch in primary outcome(s). The primary outcome was the mean number of domains accurately classified (scale from 0 to 9).

**Results:**

The mean (SD) number of domains (0 to 9) accurately classified per manuscript was 6.39 (1.49) for ECRs using COBPeer versus 5.03 (1.84) for the journal’s usual peer-review process, with a mean difference [95% CI] of 1.36 [0.88–1.84] (*p* < 0.001). Concerning secondary outcomes, the sensitivity of ECRs using COBPeer versus the usual peer-review process in detecting incompletely reported CONSORT items was 86% [95% CI 82–89] versus 20% [16–24] and in identifying a switch in primary outcome 61% [44–77] versus 11% [3–26]. The specificity of ECRs using COBPeer versus the usual process to detect incompletely reported CONSORT domains was 61% [57–65] versus 77% [74–81] and to identify a switch in primary outcome 77% [67–86] versus 98% [92–100].

**Conclusions:**

Trained ECRs using the COBPeer tool were more likely to detect inadequate reporting in RCTs than the usual peer review processes used by journals. Implementing a two-step peer-review process could help improve the quality of reporting.

**Trial registration:**

Clinical.Trials.gov NCT03119376 (Registered April, 18, 2017).

## Background

The peer-review process is a cornerstone of biomedical research [1]. It is considered the best method for helping scientific editors decide on the acceptability of a manuscript for publication and improving the quality of published reports [2]. Nevertheless, the effectiveness of this system has been questioned [3–8]. For example, peer reviewers do not consistently perform some essential tasks when evaluating the report of a randomized controlled trial (RCT) such as checking adherence to the CONSORT reporting guideline or checking trial registries to identify outcomes that are switched from the registered protocol [5].

Perhaps peer reviewers are expected to perform too many tasks [9], and simple and neglected tasks such as checking the reporting could be transferred to early career peer reviewers (ECRs) (i.e., junior researchers with no or little experience in the peer review of RCTs) [10]. CONSORT guidelines and the associated COBPeer tool have been developed with the intent of making it possible to expect that after some basic training ECRs can screen for key items in a manuscript, thereby letting the already over-burdened senior/experienced reviewers focus on the areas where their subject and technical expertise will be of most value.

The objectives of this study were to evaluate accuracy in identifying inadequate reporting (i.e., incomplete reporting and a switch in primary outcome) in two-arm parallel-group RCT reports by ECRs using the COBPeer tool versus the usual journal peer-review process.

## Methods

### Study design

We performed a cross-sectional diagnostic study to identify inadequate reporting of RCTs. The study was developed, and the results are reported according to the guidelines on the STAndards of the Reporting of Diagnostic accuracy (STARD) [11]. The checklist is available in Additional file 1. The protocol was published previously [12]. There were no major changes to the protocol (Additional file 2).

Inadequate reporting was defined as incomplete reporting or a switch in primary outcome. It considered nine domains: eight most important CONSORT domains (rated as incompletely reported, yes/no) and a switch in primary outcome(s) (rated as a switch/no switch) [5]. The eight most important CONSORT domains (which include 10 items) concern:

- Outcomes (item 6a),
- Randomization/sequence generation (item 8a),
- Allocation concealment mechanism (item 9),
- Blinding (items 11a, 11b),
- Participant flow (items 13a, 13b),
- Outcomes and estimation (item 17a),
- Harms (item 19), and
- Trial registration (item 23).

We defined a switch in primary outcome as a primary outcome was added, deleted, or changed between the primary outcome(s) published in the protocol or register and the primary outcome(s) reported in the report. Moreover, if the reference standard identified discrepancies in definition of the primary outcome(s) (i.e., variable of interest, terms of time frame, metric) between the primary outcome(s) registered and reported in the report, we considered that like a switch in primary outcome.

These domains were considered most important because they are frequently incompletely reported and are necessary for conducting a systematic review to evaluate the risk of bias and record the outcome data [5].

To assess accuracy in identifying inadequate reporting, we used two tests: (1) ECRs assessing a manuscript by using the COBPeer tool (after completing an online training module) and (2) the usual journal peer-review process (i.e., any peer reviewer assessing a manuscript as per the first round of the journal’s peer-review process). The reference standard was the assessment of the manuscript by two experienced systematic reviewers achieving consensus in case of discrepancies. Thus, we compared face-to-face the accuracy in identifying inadequate reporting by the ECRs using the COBPeer tool and by the actual peer reviewers involved in the process at the first round.

The assessment of the reports by the usual journal’s peer-review process was performed before this study was conducted. However, the data extracted from the peer-review reports produced during the journal’s usual peer-review process, as well as the assessment of the manuscript by ECRs and the reference standard were performed prospectively.

### Manuscript identification

We identified a sample of 120 manuscripts reporting the results of two-arm parallel-group RCTs for which we could access the first manuscript submitted and all peer-review reports. We searched PubMed using the “article types” filter *randomized controlled trial* to identify all articles reporting results of RCTs published between 1 January 2015 and 13 December 2016 (search date: December 14, 2016) in:

- BMC series medical journals that publish at least five RCT reports per year,
- *BMJ,*
- *BMJ Open,* and
- *Annals of Emergency Medicine*.

These journals were chosen either because the peer-review reports are available online (i.e., BMC series medical journal, *BMJ* and *BMJ Open*) or because editors gave us access to peer-review reports (i.e., *Annals of Emergency Medicine*).

The search strategy is given in Additional file 3. One researcher (AC) screened all titles and abstracts and included all reports of two-arm parallel-group RCTs assessing any intervention in human participants. We excluded cluster RCTs, cross-over trials, equivalence and non-inferiority trials, feasibility studies, cost-effectiveness studies, phase I trials, study protocols, non-RCTs, secondary publications or analyses, pilot studies, systematic reviews, methodology studies, and early-phase studies. All journals included endorsed the CONSORT statement.

For each article identified, we retrieved the manuscript submitted to the first round of the peer-review process as well as all the peer-review reports submitted by peer reviewers during this first round. If the manuscript or any peer-review reports were not available, the RCT was excluded. Overall, of the 1600 citations retrieved, 222 were eligible, and 17 were excluded because the manuscript submitted or peer-review reports were not available. Of these, we randomly selected 120 reports to be evaluated. These reports were published in 24 different journals (i.e., *BMJ*, *BMJ Open*, *Annals of Emergency Medicine*, and 22 journals of the BMC series). The reference list is in Additional file 4.

Each manuscript had to be evaluated by a single ECR using COBPeer. A single ECR was considered sufficient because the ECRs had a single task to perform and they were supported by COBPeer. Furthermore, this design should facilitate future implementation in practice.

### Test methods

#### ECRs using COBPeer after completing an online training module

##### COBPeer

The principle of the COBPeer tool is detailed elsewhere [12]. In brief, COBPeer is an online CONSORT-based peer-review tool assessing nine domains: the eight most important CONSORT domains (i.e., 10 CONSORT items) and a switch in primary outcome(s) (Additional file 5).

For the CONSORT domains, COBPeer was developed following the same principles used to develop the CONSORT-based writing aid tool CobWeb [i.e., CONSORT item(s) are elicited with bullet points extracted from the CONSORT Explanation and Elaboration Explanation paper] [13]. Bullet points specify exactly what should be reported and elicit the meaning of the item [13, 14]. ECRs using COBPeer had to indicate for each bullet point if the requested information was completely reported (yes/no). A domain was considered incompletely reported if information for at least one bullet point was missing.

For the “switched primary outcome” domain, ECRs had to compare the primary outcome(s) reported in the manuscript to that reported in the trial register based on the process used in COMPare [15] and to indicate whether there was “no switch in primary outcome,” “presence of a switch in primary outcome” (i.e., at least one primary outcome was switched), or “unable to assess” (i.e., the study was not registered or the primary outcome was not sufficiently defined). After all items are assessed, the tool automatically generates a standardized and individualized peer-review report detailing what information is missing.

To use the tool, ECRs had to complete a training module. The module starts with a short reminder about the importance of assessing completeness of reporting and switched outcomes. Then, the module goes through each item starting with a description of the item and related bullet points. ECRs have to complete assessments of two domains from published RCTs for each CONSORT domain by using the tool. For each extract assessed, ECRs receive a personalized feedback with a detailed explanation.

**Fig. 1:**
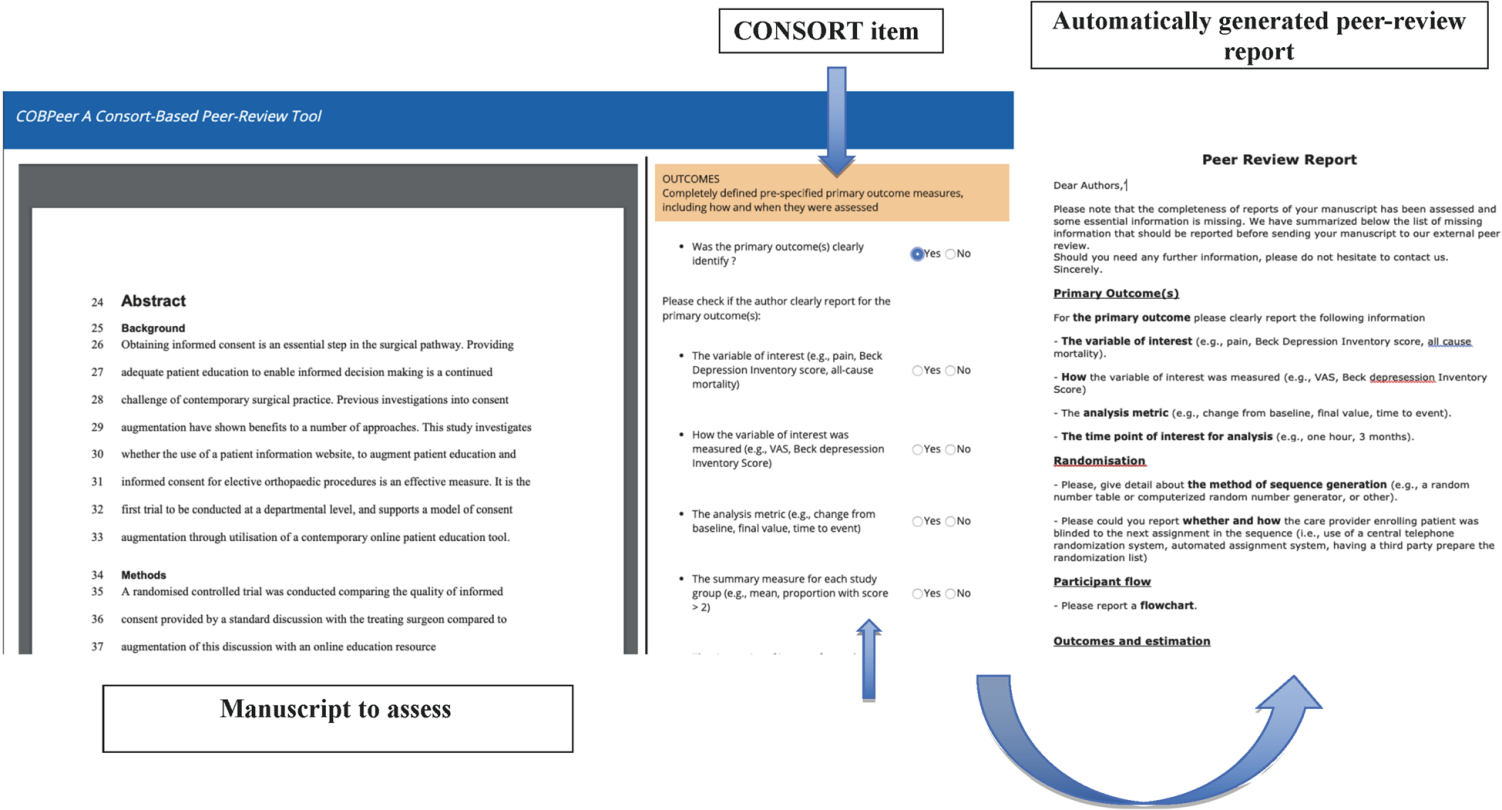
Example of the CONSORT-based peer-review tool (COBPeer)

**Fig. 2:**
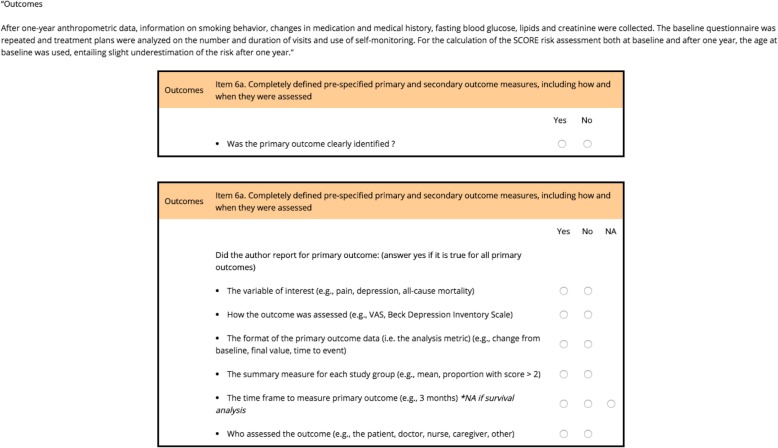
Example of the CONSORT-based peer-review tool (COBPeer)

The module, as well as COBPeer, is available from [16] and the tool is available from [17]. Examples of COBPeer and the module are in Figs. 1 and 2. The COBPeer tool is in Additional file 6.

##### ECRs

Participants were early-stage researchers (i.e., Masters students, PhD students, residents involved in clinical research during their study, and clinicians involved in clinical research) who had never peer reviewed an RCT manuscript previously.

To recruit participants, we advertised the study by contacting editors of biomedical journals (e.g., *BMC Medicine*, *Trials*, *Peer Review and Research Integrity*), learned societies (e.g., European Society of Emergency Medicine), universities (Paris Descartes and Paris Diderot), an international network of students (Students 4 Best Evidence), and our own network (Twitter). Participants were invited to participate in an academic study of an online training module and tool dedicated to supporting ECRs in assessing results of an RCT (Additional file 7). Participants accessed the COBPeer platform via an online link.

After completing the training module, ECRs who agreed to participate in the study had to evaluate one of the randomly selected RCT reports using COBPeer. They were asked to perform this assessment within 30 min maximum, although the exact duration was not monitored. Each ECR assessed one manuscript.

ECRs were blinded to the journal name, author names and study title, the aim of the study, systematic reviewers’ assessments, and peer reviewers’ comments.

#### Usual peer-review process

The peer reviewers involved in the usual peer-review process were those invited by editors who agreed to review the manuscript and submitted their peer-review report during the usual process. They were invited and accepted before the study was planned and they were consequently not aware of the aim of this study and were blinded to the assessment by the reference standard and ECRs. For each manuscript identified, we retrieved and merged all the peer-review reports available after the first round for peer review.

Two senior clinical epidemiologists independently extracted data from the peer-review report by using a standardized data extraction form available in Additional file 8. These researchers were not involved in other data extraction. They were blinded to the reference standard and ECR assessments. Disagreements were discussed to achieve consensus.

The researchers recorded whether the peer reviewers raised some concerns about the completeness of reporting of the eight CONSORT domains considered and identified a switch in primary outcome between the manuscript and the register. The assessments in all peer-review reports for each manuscript were combined, and the domain was rated as “incompletely reported” or “presence of switch in primary outcome” if at least one peer reviewer rated it as such. In case of disagreement between peer reviewers (one raising concerns about the completeness of reporting and one highlighting the adequacy of reporting), the domain was rated as “incompletely reported” or “presence of switch in primary outcome.” Any discrepancies were discussed to achieve consensus.

#### Reference standard

The reference standard was the assessment of the manuscript by four pairs of systematic reviewers with expertise in the conduct of systematic reviews. They independently evaluated the completeness of reporting of the eight CONSORT domains by using the CONSORT checklist (i.e., domains with CONSORT items provided without bullet points). They rated each domain as “completely reported,” “partially reported,” or “not reported.” A domain was considered incompletely reported if it was rated “partially reported” or “not reported.” The systematic reviewers were also asked to systematically compare the primary outcome reported in the manuscript and the primary outcome reported in the registry and indicate “no switch in primary outcome,” “presence of a switch in primary outcome,” “not available” (i.e., not registered), or “unable to assess” (i.e., insufficiently described in register). We defined switch in primary outcome as a primary outcome was added, deleted, or changed between the primary outcome(s) published in the protocol or register and the primary outcome(s) reported in the report. Moreover, if reference standard identified a discrepancy in definition of the primary outcome(s) (i.e., variable of interest, terms of time frame, metric) between the primary outcome(s) registered and reported in the report, we considered that like a switch in primary outcome.

This assessment corresponded to the assessment of the risk of bias (random sequence generation, allocation concealment, blinding of participants and personnel, blinding of outcome assessment, selective reporting of outcomes) and the extraction of efficacy and harms outcome data in a systematic review.

The systematic reviewers were asked to rate domains as incompletely reported only if the reporting was a real barrier to the conduct of a systematic review. This approach allows for a reference standard evaluation from systematic reviewers’ perspectives and avoids focusing on the reporting of useless details (Table 1).

**Table 1:** Assessment of domains by usual peer reviewer, reference standard, and early career peer reviewer

Usual peer reviewer	➢ For CONSORT domains: For each manuscript included, determine whether the peer reviewers and/or editors raised some concern on the completeness of reporting of the following CONSORT items. The assessment of all peer-review reports and editors’ comments for each manuscript need to be combined. - Yes, some concern was raised - No, some concern was not raised ➢ For switched outcomes: For each manuscript included, check whether peer reviewers and/or editors identified inconsistency between data registered and reported for the primary outcome(s). - Yes, inconsistency was detected - No, inconsistency was not detected - Not available, because the study was not registered or the protocol was not available Comments: For blinding domains researchers could quote “unblinded study.” Moreover, if the domain was considered partially reported or not reported, it was quoted as not reported.
Reference standard	➢ For CONSORT domains: Now you will have to evaluate in each RCT if authors correctly reported all key elements of selected CONSORT items. Please evaluate whether authors correctly reported all key elements of the domain considered. Rate items as inadequately reported only if the reporting is a real barrier to the conduct of a systematic review. - Completely reported - Partially reported - Not reported ➢ For switched outcomes: Did authors register their protocol after the beginning of the study? - Yes - No - Not available Inconsistency between data registered and reported for the primary outcome(s) (i.e., at least one primary outcome added, deleted, or changed)? - Yes - No - Not available, because the study was not registered or the protocol is not available - Unable to assess (i.e., outcomes insufficiently described in the register) Comments: For blinding domains researchers could quote “not available because blinding was impossible.” Moreover, domains partially reported or not reported were quoted as not reported.
Early career peer reviewer	See Additional file 5

The systematic reviewers were blinded to the peer-reviewer assessments, ECR assessments, and the content of COBPeer. They were instructed to base their assessment only on the content of the manuscript. Any differences between systematic reviewers were resolved by discussion, with the involvement of an arbitrator if necessary.

### Outcomes

The primary outcome was the mean number of domains accurately classified per manuscript initially submitted to the journal on a scale from 0 to 9. Each of the eight CONSORT domains was rated “incompletely reported” (yes/no); when blinding was not possible, the related domain (item 11a/b) was rated “no incomplete reporting.” The domain on “switch primary outcome” was rated “yes/no” or “unable to assess or unavailable.” Table 1 describes the modalities of assessment and cutoff for each test and reference standard.

Secondary outcomes were the mean number of CONSORT domains accurately classified per manuscript and the sensitivity, specificity, and likelihood ratio to detect the domains as incompletely reported and to identify a switch in primary outcome.

We also performed a sensitivity analysis to check that our results were not related to the reference standard. For this purpose, for all false-positive domains (i.e., the domain was considered not adequately reported by ECRs using COBPeer or by the usual peer-review process, but the reference standard considered the domain adequately reported), we asked the two systematic reviewers to check their assessment. They could change their assessment if necessary. They were blinded to whether the false positive was identified by ECRs or the usual peer-review process.

### Sample size calculation

We allowed for detecting an effect size of 0.3 for the mean number of domains accurately classified per manuscript with a power of 90% and a two-sided alpha level of 5%. To that end, we needed evaluation of 120 reports [18]. Each ECR included, assessed 1 manuscript, so we randomly selected 120 reports of two-arm parallel-group RCTs to be assessed in the study. We analyzed only the first evaluation of each report.

### Statistical analysis

Quantitative data are described with means (SD) and/or medians [Q1-Q3] and categorical data with numbers (%). We compared the mean number of domains accurately classified per manuscript by ECRs versus the usual peer-review process by using paired *t* test. The sensitivity and specificity for the ECRs and the usual peer-review process were compared by using an exact McNemar chi-square test. Exact binomial 95% confidence intervals (CIs) were calculated for sensitivity and specificity [19]. Positive and negative likelihood ratios were computed, and 95% CIs were based on formulae provided by Simel et al. [20]. *SAS 9.4* (*SAS* Institute *Inc., Cary*, *NC*) was used for descriptive statistics and tests and the epiR package in R v3.5.1 to estimate diagnostic performance parameters [21].

### Changes to the protocol

There were no changes in the protocol, but the wording used was slightly modified and clarified.

First, we clarified that we focused on the eight most important CONSORT domains (which include 10 CONSORT items). Second, we classified each item as “incompletely reported” (yes/no) instead of “completely reported” (yes/no) as stated in the protocol and registry. Third, we clarified the assessment of domains by usual peer reviewers, reference standard, and early career peer reviewers in Table 1 which was not completely detailed in the protocol and registry.

## Results

### Manuscript characteristics

Of the 120 reports selected, 1 was not evaluated because of a technical issue in the online randomization system. The report was consequently excluded. The 119 reports of two-arm parallel-group RCTs were published in 24 different journals. The most frequently tested intervention was drug (*n* = 55/119; 46%) (Additional file 9). The mean (SD) number of peer reviewers per manuscript involved in the initial peer review assessment was 2.5 (1.0) (range 1-8).

#### ECR characteristics

In total, 167 participants registered online to access the training module; 131 (78%) performed the training module and peer reviewed the allocated manuscript; and 119 were included in the analysis (i.e., the first assessment of the 119 identified manuscripts) to adhere to the planned sample size (Additional file 4).

Participant characteristics are in Table 2. Participants were mainly located in France (44%), USA/Canada (32%), UK (12%), and other European countries (10%). Most participants were physicians (89%); 41% (*n* = 49) had a Master of Science degree and 27% (*n* = 32) were PhD candidates. Two thirds were male (*n* = 72; 61%). Only 16% (*n* = 19) were trained to perform a peer review but two thirds (*n* = 77; 65%) had received some training on critical assessment of RCT reports.

**Table 2:** General characteristics of early career peer reviewers (ECRs) participating in the study (*n* = 119)

Sex	
Male	72 (60.5)
Country	
France	52 (43.7)
USA	20 (16.8)
Canada	18 (15.1)
UK	14 (11.8)
Other European country	12 (10.1)
South America	2 (1.7)
Africa	1 (0.8)
Professional background	
Physician	106 (89.1)
Student	11 (9.2)
Other	2 (1.7)
Academic background	
Master of Science	49 (41.2)
Doctor of Medicine (MD)	36 (30.2)
PhD	32 (26.9)
Other	2 (1.7)
How did you hear about this training program?	
Faculty	84 (70.6)
Social network	11 (9.2)
Network of international students	7 (5.9)
Editors of biomedical journals	2 (1.7)
Learned societies	2 (1.7)
Other	13 (10.9)
Previously trained to perform a peer review	19 (16.0)
Previously trained to appraise an RCT report	77 (64.7)

Data are *n* (%)

*RCT* randomized controlled trial

Table 3 shows the results for participants during the training module. The mean (SD) percentage of correct answers during training was 60 (23). The domain less accurately evaluated by the participants was participant flow (items 13a/13b) (*n* = 77/238 assessments, 32%). The domain most accurately evaluated by the participants was trial registration (item 23) (*n* = 238/238 assessments, 100%).

**Table 3:** Results for ECRs when performing the training module. The module contained two extracts per domains that the ECR had to evaluate. The answer was considered appropriate when all bullet points were correctly assessed

Items assessed	Percentage of correct answers *n*/*N* (%)
- Item 6a (Outcomes)	83/238 (34.9)
- Item 8a (Randomization/sequence generation)^α^	221/237 (92.3)
- Item 9 (Allocation concealment mechanism)	186/238 (78.2)
- Item 11a/b (Blinding)	107/238 (45.0)
- Item 13a/b (Participant flow)	77/238 (32.4)
- Item 17a (Outcomes and estimation)	152/238 (63.9)
- Item 19 (Harms)^β^	97/238 (41.1)
- Item 23 (Trial registration)	238/238 (100.0)
Switch in primary outcomes^δ^	
- Outcome(s) reported by the authors as primary outcome(s) while not registered as such	116/198 (58.6)
- Outcome(s) registered as primary outcome but not reported as such in the manuscript	103/198 (52.0)

Data are *n* (%)

^α^One missing value, ^β^Two missing values, ^δ^40 missing values. All missing values due because of a technical issue

#### Outcomes

##### Accuracy in detecting inadequate reporting

After consensus between the two systematic reviewers, the reference standard revealed that for the 119 selected manuscripts, domains were incompletely reported in 15% (*n* = 18/119) (item 23, trial registration) to 60% (*n* = 71/119) (item 19, harms) of manuscripts, and 30% (*n* = 36/119) of the manuscripts had switch in primary outcome. Table 4 reports the details for each domain.

**Table 4:** Completeness of reporting and a switch in primary outcome as rated by the reference standard (two systematic reviewers) for the sample of 119 manuscripts

CONSORT items incompletely reported	*N* = 119 (%)
- Item 6a (Outcomes)	58 (48.7)
- Item 8a (Randomization/sequence generation)	38 (31.9)
- Item 9 (Allocation concealment mechanism)	62 (52.1)
- Items 11a/11b (Blinding)	51 (42.9)
- Items 13a/13b (Participant flow)	39 (32.8)
- Item 17a (Outcomes and estimation)	48 (40.3)
- Item 19 (Harms)	71 (59.7)
- Item 23 (Trial registration)	18 (15.1)
Switch in primary outcomes	
- Yes	36 (30.3)

Data are *n* (%)

Considering the nine domains of the tool (eight CONSORT domains and one domain for a switch in primary outcome), the mean (SD) number of domains accurately classified per manuscript was 6.39 (1.49) for ECRs using COBPeer versus 5.03 (1.84) for the journal’s usual peer-review process, with a mean difference [95% CI] of 1.36 [0.88–1.84] (*p* < 0.001) (Table 5).

**Table 5:** Accuracy of ECRs using COBPeer and usual peer review in detecting inadequate reporting (i.e., items completely reported or a switch in primary outcome) by the reference standard (two systematic reviewers). ECR had to evaluate 9 domains in 119 manuscripts, therefore 1071 items

Inadequate reporting identified	Reference standard	
	Yes *N* = 421	No *N* = 650
ECRs		
Yes	352	241
No	69	409
Usual peer review		
Yes	80	131
No	341	519

##### Secondary outcomes

The mean (SD) number of domains accurately classified per manuscript for the eight CONSORT domains was 5.67 (1.41) for ECRs using COBPeer versus 4.32 (1.81) for the journal’s usual peer-review process, with a mean difference of 1.36 [0.88–1.84]; *p* < 0.001.

Sensitivity, specificity, and positive and negative likelihood ratios for each domain are in Table 6. Sensitivity of ECRs versus the journal’s usual peer-review process was 86% [95% CI 82–89] versus 20% [16–24] to detect incompletely reported CONSORT domains and 61% [44–77] versus 11% [3–26] to identify a switch in primary outcome. Specificity of ECRs versus the usual process to detect incompletely reported CONSORT domains was 61% [57–65] versus 77% [74–81] and to identify a switch in primary outcome 77% [67–86] versus 98% [92–100].

**Table 6:** Sensitivity, specificity, positive likelihood ratio, and negative likelihood ratio for usual peer review and ECRs using COBPeer for each item

	Sensitivity (%)			Specificity (%)			Positive likelihood ratio		Negative likelihood ratio	
	ECR	Usual peer review	*p* value	ECR	Usual peer review	*p* value	ECR	Usual peer review	ECR	Usual peer review
All domains	83.6 [79.7–87.0]	19.0 [15.4–23.1]	< 0.001	62.9 [59.1–66.6]	79.9 [76.6–82.9]	< 0.001	2.26 [2.02–2.51]	0.94 [0.73–1.21]	0.26 [0.21–0.33]	1.01 [0.96–1.08]
All CONSORT domains	85.7 [81.8–89.1]	19.7 [15.9–24.1]	< 0.001	60.9 [56.7–64.9]	77.2 [73.6–80.6]	< 0.001	2.19 [1.96–2.44]	0.87 [0.67–1.12]	0.23 [0.18–0.30]	1.04 [0.97–1.11]
- Item 6a (Outcomes)	94.8 [85.6–98.9]	27.6 [16.7–40.9]	< 0.001	42.6 [30.0–55.9]	57.4 [44.1–70.0]	0.16	1.65 [1.32–2.07]	0.65 [0.39–1.08]	0.12 [0.04–0.38]	1.26 [0.97–1.65]
- Item 8a (Randomization/sequence generation)	76.3 [59.8–88.6]	34.2 [19.6–51.4]	< 0.001	85.2 [75.6–92.1]	76.5 [65.8–85.2]	0.21	5.15 [2.97–8.94]	1.46 [0.81–2.63]	0.28 [0.16–0.50]	0.86 [0.66–1.11]
- Item 9 (Allocation concealment mechanism)	82.3 [70.5–90.8]	8.1 [2.7–17.8]	< 0.001	71.9 [58.5–83.0]	87.7 [76.3–94.9]	0.05	2.93 [1.90–4.51]	0.66 [0.22–1.95]	0.25 [0.14–0.43]	1.04 [0.93–1.18]
- Items 11/1b (Blinding)	56.9 [42.2–70.7]	19.6 [9.8–33.1]	< 0.001	76.5 [64.6–85.9]	83.8 [72.9–91.6]	0.38	2.42 [1.48–3.95]	1.21 [0.56–2.63]	0.56 [0.40–0.79]	0.96 [0.81–1.14]
- Items 13a/13b (Participant flow)	92.3 [79.1–98.3]	17.9 [7.5–33.5]	< 0.001	21.3 [12.9–31.8]	78.8 [68.2–87.1]	< 0.001	1.17 [1.01–1.36]	0.84 [0.38–1.87]	0.36 [0.11–1.16]	1.04 [0.87–1.25]
- Item 17a (Outcomes and estimation)	93.8 [82.8–98.7]	33.3 [20.4–48.4]	< 0.001	56.3 [44.0–68.1]	64.8 [52.5–75.8]	0.42	2.15 [1.63–2.82]	0.95 [0.57–1.58]	0.11 [0.04–0.34]	1.03 [0.79–1.34]
- Item 19 (Harms)	97.2 [90.2–99.7]	9.9 [4.1–19.3]	< 0.001	8.3 [2.3–20.0]	75.0 [60.4–86.3]	< 0.001	1.06 [0.97–1.16]	0.39 [0.17–0.93]	0.34 [0.06–1.77]	1.20 [1.00–1.44]
- Item 23 (Trial registration)	88.9 [65.3–98.6]	11.1 [1.4–34.7]	< 0.001	95.1 [88.8–98.4]	88.1 [80.2–93.7]	0.09	17.96 [7.52–42.86]	0.94 [0.23–3.83]	0.12 [0.03–0.43]	1.01 [0.84–1.21]
Switched outcomes	61.1 [43.4–76.9]	11.1 [3.1–26.1]	< 0.001	77.1 [66.6–85.6]	97.6 [91.6–99.7]	< 0.001	2.67 [1.66–4.28]	4.61 [0.88–24.05]	0.50 [0.33–0.77]	0.91 [0.81–1.03]

**Fig. 3:**
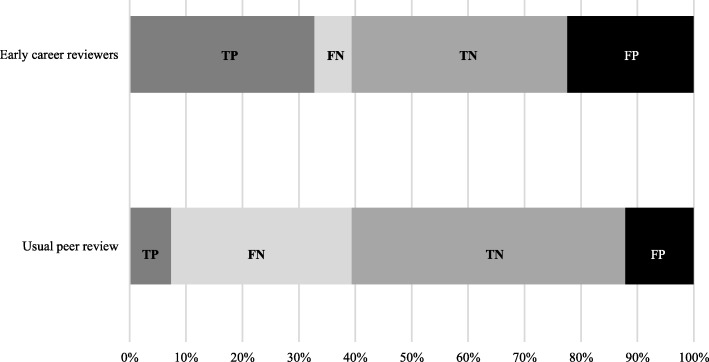
Proportions of items evaluated by early career reviewers and usual peer review classified as true positive (TP), false negative (FN), true negative (TN), and false positive (FP)

Figure 3 shows the proportions of items evaluated by ECRs and the journal’s usual peer-review process classified as true positive, false negative, true negative, and false positive.

### Sensitivity analysis

Among the 372 false-positive domains, only eight assessments (2.2%) were reclassified by the reference standard, one for blinding (items 11a/11b) and seven for participant flow (items 13a/13b). The sensitivity analysis showed that the mean (SD) number of domains accurately classified per manuscript initially submitted to the journal was 6.39 (1.49) for ECRs using COBPeer versus 5.03 (1.84) for the journal’s usual peer-review process, with a mean difference [95% CI] of 1.36 [0.88–1.84]; *p* < 0.001. Additional file 10 reports results of the sensitivity analysis after exploring false-positive results.

## Discussion

In this study, we explored a new approach for the peer-review process. We proposed to transfer some tasks that are essential but often neglected by peer reviewers to early career researchers who had not previously been involved in peer review. For this purpose, we focused on a small number of relatively simple tasks (evaluating adherence to eight CONSORT domains and identifying a switch in primary outcome). To standardize this task and the feedback provided to authors, we developed the COBPeer tool, a specific online tool and a training module. ECRs using COBPeer were more accurate than the journal’s usual peer-review process in detecting inadequate reporting. They showed high sensitivity but lower specificity in detecting incomplete reporting and a switch in primary outcome.

The interpretation of these results should consider differences between the different processes. First, ECRs were prompted and supported by COBPeer to check the completeness of reporting of the eight CONSORT domains and a switch in outcome; it was the only task they had to do, and they were specifically trained to do it. In contrast, usual peer reviewers were not specifically prompted to check the adequacy of reporting, they did not have access to COBPeer, and they were requested to perform several tasks other than checking the reporting. Nevertheless, because we selected journals endorsing CONSORT, peer reviewers may more likely be aware of issues of transparency.

Furthermore, ECRs were aware that they were participating in a research project, whereas usual peer reviewers did not know that their assessment would be used in a study on the completeness of reporting. However, because the peer-review process was an open process, peer reviewers could be encouraged to perform a more complete review knowing that it was made publicly available.

Finally, the results were based on the assessment by only one ECR using COBPeer per manuscript, whereas for the usual peer-review process, we considered the assessment of all peer reviewers involved in the first round (i.e., 2.5, on average).

This study has important strengths. We identified a large sample of manuscripts submitted to 24 journals; the peer-review process was conducted as usual according to each journal’s strategy, and the information provided to peer reviewers was not modified because we retrospectively evaluated a manuscript’s peer-review report. We had a large recruitment strategy for ECRs, who came from various countries. Our approach related to the reference standard was pragmatic to avoid considering some domains as incompletely reported, and the information provided is sufficiently detailed to be integrated in a systematic review.

Our study has some limitations. First, we focused on only two-arm parallel-group RCTs and cannot extrapolate our findings to other more complex study designs. But this design is the most popular and reported one. Second, almost all of the ECRs were physicians and this may not reflect the broad spectrum of peer reviewers. Third, we included only articles that were peer reviewed and published. This inclusion may imply that the quality of the methods and reporting of these manuscripts was probably initially relatively good and may not reflect the quality of all submitted manuscripts. Fourth, we considered only the first round of the peer-review process and cannot exclude that some peer reviewers identified inadequate reporting at a later stage of the process. However, improving transparency should be a task performed at an early stage in the process because lack of transparency is a major barrier to an accurate evaluation of methodological quality. Fifth, the online tool considered only eight CONSORT domains and a switch in primary outcome. However, we focused on the most important and often poorly reported domains. Lastly, we did not select ECRs according to their results after the training module. We could expect that deciding a threshold for authorization to become an ECR reviewer would improve the results.

The results of this study have important implications. First, we could implement a new process of peer-review relying on a two-stage peer review. However, we need to explore the feasibility of this process. The involvement of junior reviewers could have an impact on the whole peer-review system. This new system could also increase the duration of the peer-review process and increase the burden on authors. We need to evaluate this new system in an RCT. Finally, we believe that COBPeer could probably be used by stakeholders other than early career researchers. According to the in-house support available for editors, COBPeer could probably be used by editorial managers or other staff.

## Conclusions

Our study showed that trained ECRs using the COBPeer tool were more accurate than processes used by journal in detecting inadequate reporting in reports of two-arm parallel-group RCTs. A new two-step peer-review process could help increase the number of peer reviewers involved and improve the quality of reporting manuscripts sent to senior peer reviewers as well as facilitate and improve the quality of the tasks performed by senior peer reviewers. This new process should nevertheless be evaluated.

## Supplementary information

**Additional file 1.** STARD checklist. Full details of the reporting according the checklist.

**Additional file 2.** Minor changes to the protocol. Details of the wording used to describe the primary outcome that was slightly modified.

**Additional file 3.** The search strategy in Medline via PubMed. Full list of search terms and filters.

**Additional file 4.** The 120 randomized controlled trials included in our study. Due to a technical issue the manuscript number 63 was not evaluated by early career reviewer. Full references of manuscript included in the study.

**Additional file 5.** The summary of the process. Full details of the design of the COBPeer study.

**Additional file 6.** The COBPeer tool. Details of each domains evaluated by early career researchers with COBPeer tool.

**Additional file 7.** Information for participants. The invitation letter received by the early career researchers.

**Additional file 8.** Screenshot of the content of the standardized data extraction form used for the evaluation of the usual peer-review. Example of the data extraction form used.

**Additional file 9.** General characteristics of reports of randomized controlled trials included in the study. Details of reports included in the study.

**Additional file 10.** Sensitivity analysis after exploring false-positive results. Analysis after reclassification of false-positive results by the reference standard.

## Data Availability

The datasets used and/or analyzed during the current study are available from the corresponding author on reasonable request.
