# Supplementary material for: Accuracy in detecting inadequate research reporting by early career peer reviewers using an online CONSORT-based peer-review tool (COBPeer) versus the usual peer-review process: a cross-sectional diagnostic study
Source: BMC Med. 2019 Nov 19;17:205. doi: 10.1186/s12916-019-1436-0 (PMC6864983; doi:10.1186/s12916-019-1436-0)
Supplement: Supplementary file 2 — Additional file 2. Minor changes to the protocol. Details of the wording used to describe the primary outcome that was slightly modified. [file 12916_2019_1436_MOESM2_ESM.docx]

|  |  |  |  |  |
| --- | --- | --- | --- | --- |

Additional file 2. Minor changes to the protocol

The wording used to describe the primary outcome was slightly modified and the changes are detailed below

| Protocol | Registry | Article |
| --- | --- | --- |
| We focus on the 10 CONSORT items which are classified in 8 domains | We focus on the 10 CONSORT items which are classified in 8 domains | To avoid confusion, we focus on the 8 CONSORT domains which include the 10 CONSORT items |
| The wording for the answer modalities was “adequately reported” (yes/no) | The wording for the answer modalities was “adequately reported” (yes/no) | The wording for the answer modalities was changed to “incompletely reported” (yes/no) to be consistent with the last domain focusing on “presence of a switch in primary outcomes” (yes/no) |
| The threshold for each test was not clearly defined | The threshold for each test was not clearly defined | The threshold is now clearly defined |
