# Supplementary material for: Accuracy in detecting inadequate research reporting by early career peer reviewers using an online CONSORT-based peer-review tool (COBPeer) versus the usual peer-review process: a cross-sectional diagnostic study
Source: BMC Med. 2019 Nov 19;17:205. doi: 10.1186/s12916-019-1436-0 (PMC6864983; doi:10.1186/s12916-019-1436-0)
Supplement: Supplementary file 3 — Additional file 3. The search strategy in Medline via PubMed. Full list of search terms and filters. [file 12916_2019_1436_MOESM3_ESM.docx]

Additional file 3. The search strategy in Medline via PubMed

Search date: December 14, 2016

Article types filter: *randomized controlled trial*

Publication date filter: between 1 January 2015 and 13 December 2016

Equation: “BMJ” [Journal] OR “BMJ open” [Journal] OR “Ann Emerg Med” [Journal] OR “Arch Public Health” [Journal] OR “BMC Anesthesiol” [Journal] OR “BMC Cancer” [Journal] OR “BMC Cardiovasc Disord” [Journal] OR “BMC Clin pathol” [Journal] OR “BMC Complement Altern Med” [Journal] OR “BMC dermatol” [Journal] OR “BMC Ear Nose Throat Disord” [Journal] OR “BMC emerg med” [Journal] OR “BMC endocr disord” [Journal] OR “BMC Fam Pract” [Journal] OR “BMC Gastroenterol” [Journal] OR “BMC Geriatr” [Journal] OR “BMC Hematol” [Journal] OR “BMC Infect Dis” [Journal] OR “BMC Int Health Hum Rights” [Journal] OR “BMC Med Educ” [Journal] OR “BMC Med Imaging” [Journal] OR “BMC Med Inform Decis Mak” [Journal] OR “BMC Med” [Journal] OR “BMC Musculoskelet Disord” [Journal] OR “BMC Nephrol” [Journal] OR “BMC Neurol” [Journal] OR “BMC Nurs” [Journal] OR “Nutr J” [Journal] OR “BMC obes” [Journal] OR “BMC ophtalmol” [Journal] OR “BMC oral health” [Journal] OR “BMC palliative care” [Journal] OR “BMC Pediatr” [Journal] OR “BMC Pharmacol Toxicol” [Journal] OR “BMC Pregnancy Childbirth” [Journal] OR “BMC Public Health” [Journal] OR “BMC Psychiatry” [Journal] OR “BMC Psychol” [Journal] OR “BMC Pulm Med” [Journal] OR “BMC Sports Sci Med Rehabil” [Journal] OR “BMC Surg” [Journal] OR “BMC urol” [Journal] OR “BMC Womens Health” [Journal] OR “Cardiovasc ultrasound” [Journal] OR “environ health” [Journal] OR “Head face Med” [Journal] OR “Hered Cancer Clin Pract” [Journal] OR “Implement Sci” [Journal] OR “J Cardiothorac Surg” [Journal] OR “J Foot Ankle Res” [Journal] OR “J Negat Results Biomed” [Journal] OR “Reprod Health” [Journal] OR “Scoliosis Spinal Disord” [Journal] OR “Syst rev” [Journal] OR “trials” [Journal]
