# Supplementary material for: Accuracy in detecting inadequate research reporting by early career peer reviewers using an online CONSORT-based peer-review tool (COBPeer) versus the usual peer-review process: a cross-sectional diagnostic study
Source: BMC Med. 2019 Nov 19;17:205. doi: 10.1186/s12916-019-1436-0 (PMC6864983; doi:10.1186/s12916-019-1436-0)
Supplement: Supplementary file 4 — Additional file 4. The 120 randomized controlled trials included in our study. Due to a technical issue the manuscript number 63 was not evaluated by early career reviewer. Full references of manuscript included in the study. [file 12916_2019_1436_MOESM4_ESM.docx]

Additional file 4. The 120 randomized controlled trials included in our study. Due to a technical issue the manuscript number 63 was not evaluated by early career reviewer.

1. [Albert N](https://www-ncbi-nlm-nih-gov.gate2.inist.fr/pubmed/?term=Albert%20N%5BAuthor%5D&cauthor=true&cauthor_uid=28082379), [Melau M](https://www-ncbi-nlm-nih-gov.gate2.inist.fr/pubmed/?term=Melau%20M%5BAuthor%5D&cauthor=true&cauthor_uid=28082379), [Jensen H](https://www-ncbi-nlm-nih-gov.gate2.inist.fr/pubmed/?term=Jensen%20H%5BAuthor%5D&cauthor=true&cauthor_uid=28082379), et al. Five years of specialised early intervention versus two years of specialized early intervention followed by three years of standard treatment for patients with a first episode psychosis: randomised, superiority, parallel group trial in Denmark (OPUS II). [BMJ.](https://www-ncbi-nlm-nih-gov.gate2.inist.fr/pubmed/28082379) 2017 Jan 12;356:i6681

2. [Amer MM](https://www-ncbi-nlm-nih-gov.gate2.inist.fr/pubmed/?term=Amer%20MM%5BAuthor%5D&cauthor=true&cauthor_uid=26395085), [Abdelaal Ahmed Mahmoud A](https://www-ncbi-nlm-nih-gov.gate2.inist.fr/pubmed/?term=Abdelaal%20Ahmed%20Mahmoud%20A%5BAuthor%5D&cauthor=true&cauthor_uid=26395085), [Abdelrahman Mohammed MK](https://www-ncbi-nlm-nih-gov.gate2.inist.fr/pubmed/?term=Abdelrahman%20Mohammed%20MK%5BAuthor%5D&cauthor=true&cauthor_uid=26395085), et al. Effect of magnesium sulphate on bi-spectral index (BIS) values during general anesthesia in children. [BMC Anesthesiol.](https://www-ncbi-nlm-nih-gov.gate2.inist.fr/pubmed/?term=26395085) 2015 Sep 22;15:126

3. [Andrade-Dávila VF](https://www-ncbi-nlm-nih-gov.gate2.inist.fr/pubmed/?term=Andrade-D%C3%A1vila%20VF%5BAuthor%5D&cauthor=true&cauthor_uid=26195123), [Chávez-Tostado M](https://www-ncbi-nlm-nih-gov.gate2.inist.fr/pubmed/?term=Ch%C3%A1vez-Tostado%20M%5BAuthor%5D&cauthor=true&cauthor_uid=26195123), [Dávalos-Cobián C](https://www-ncbi-nlm-nih-gov.gate2.inist.fr/pubmed/?term=D%C3%A1valos-Cobi%C3%A1n%20C%5BAuthor%5D&cauthor=true&cauthor_uid=26195123), et al. Rectal indomethacin versus placebo to reduce the incidence of pancreatitis after endoscopic retrograde cholangiopancreatography: results of a controlled clinical trial. B[MC Gastroenterol.](https://www-ncbi-nlm-nih-gov.gate2.inist.fr/pubmed/?term=26195123%22) 2015 Jul 21;15:85

4. [Ardigo S](https://www-ncbi-nlm-nih-gov.gate2.inist.fr/pubmed/?term=Ardigo%20S%5BAuthor%5D&cauthor=true&cauthor_uid=26767506), [Herrmann FR](https://www-ncbi-nlm-nih-gov.gate2.inist.fr/pubmed/?term=Herrmann%20FR%5BAuthor%5D&cauthor=true&cauthor_uid=26767506), [Moret V](https://www-ncbi-nlm-nih-gov.gate2.inist.fr/pubmed/?term=Moret%20V%5BAuthor%5D&cauthor=true&cauthor_uid=26767506), et al. Hypnosis can reduce pain in hospitalized older patients: a randomized controlled study. [BMC Geriatr.](https://www-ncbi-nlm-nih-gov.gate2.inist.fr/pubmed/?term=26767506) 2016 Jan 15;16:14

5. [Arora S](https://www-ncbi-nlm-nih-gov.gate2.inist.fr/pubmed/?term=Arora%20S%5BAuthor%5D&cauthor=true&cauthor_uid=24225332), [Peters AL](https://www-ncbi-nlm-nih-gov.gate2.inist.fr/pubmed/?term=Peters%20AL%5BAuthor%5D&cauthor=true&cauthor_uid=24225332), [Burner E](https://www-ncbi-nlm-nih-gov.gate2.inist.fr/pubmed/?term=Burner%20E%5BAuthor%5D&cauthor=true&cauthor_uid=24225332), [Lam CN](https://www-ncbi-nlm-nih-gov.gate2.inist.fr/pubmed/?term=Lam%20CN%5BAuthor%5D&cauthor=true&cauthor_uid=24225332), [Menchine M](https://www-ncbi-nlm-nih-gov.gate2.inist.fr/pubmed/?term=Menchine%20M%5BAuthor%5D&cauthor=true&cauthor_uid=24225332). Trial to examine text message-based mHealth in emergency department patients with diabetes (TExT-MED): a randomized controlled trial. [Ann Emerg Med.](https://www-ncbi-nlm-nih-gov.gate2.inist.fr/pubmed/?term=24225332) 2014 Jun;63(6):745-54.e6

6. [Arriens C](https://www-ncbi-nlm-nih-gov.gate2.inist.fr/pubmed/?term=Arriens%20C%5BAuthor%5D&cauthor=true&cauthor_uid=26283629), [Hynan LS](https://www-ncbi-nlm-nih-gov.gate2.inist.fr/pubmed/?term=Hynan%20LS%5BAuthor%5D&cauthor=true&cauthor_uid=26283629), [Lerman RH](https://www-ncbi-nlm-nih-gov.gate2.inist.fr/pubmed/?term=Lerman%20RH%5BAuthor%5D&cauthor=true&cauthor_uid=26283629), et al. Placebo-controlled randomized clinical trial of fish oil's impact on fatigue, quality of life, and disease activity in Systemic Lupus Erythematosus. [Nutr J.](https://www-ncbi-nlm-nih-gov.gate2.inist.fr/pubmed/?term=26283629) 2015 Aug 18;14:82

7. [Asouhidou I](https://www-ncbi-nlm-nih-gov.gate2.inist.fr/pubmed/?term=Asouhidou%20I%5BAuthor%5D&cauthor=true&cauthor_uid=26615516), [Trikoupi A](https://www-ncbi-nlm-nih-gov.gate2.inist.fr/pubmed/?term=Trikoupi%20A%5BAuthor%5D&cauthor=true&cauthor_uid=26615516). Esmolol reduces anesthetic requirements thereby facilitating early extubation; a prospective controlled study in patients undergoing intracranial surgery. [BMC Anesthesiol.](https://www-ncbi-nlm-nih-gov.gate2.inist.fr/pubmed/?term=26615516%22) 2015 Nov 28;15:172

8. [Aung EE](https://www-ncbi-nlm-nih-gov.gate2.inist.fr/pubmed/?term=Aung%20EE%5BAuthor%5D&cauthor=true&cauthor_uid=25622725), [Ueno M](https://www-ncbi-nlm-nih-gov.gate2.inist.fr/pubmed/?term=Ueno%20M%5BAuthor%5D&cauthor=true&cauthor_uid=25622725), [Zaitsu T](https://www-ncbi-nlm-nih-gov.gate2.inist.fr/pubmed/?term=Zaitsu%20T%5BAuthor%5D&cauthor=true&cauthor_uid=25622725), et al. Effectiveness of three oral hygiene regimens on oral malodor reduction: a randomized clinical trial. [Trials.](https://www-ncbi-nlm-nih-gov.gate2.inist.fr/pubmed/?term=25622725) 2015 Jan 27;16:31

9. [Ayoub N](https://www-ncbi-nlm-nih-gov.gate2.inist.fr/pubmed/?term=Ayoub%20N%5BAuthor%5D&cauthor=true&cauthor_uid=24716651), [Ghassemi A](https://www-ncbi-nlm-nih-gov.gate2.inist.fr/pubmed/?term=Ghassemi%20A%5BAuthor%5D&cauthor=true&cauthor_uid=24716651), [Rana M](https://www-ncbi-nlm-nih-gov.gate2.inist.fr/pubmed/?term=Rana%20M%5BAuthor%5D&cauthor=true&cauthor_uid=24716651), et al. Evaluation of computer-assisted mandibular reconstruction with vascularized iliac crest bone graft compared to conventional surgery: A randomized prospective study. [Trials.](https://www-ncbi-nlm-nih-gov.gate2.inist.fr/pubmed/?term=Evaluation+of+computer-assisted+mandibular+reconstruction+with+vascularized+iliac+crest+bone+graft+compared+to+conventional+surgery%3A+A+randomized+prospective+study) 2014 Apr 9;15:114

10. [Bahrami MH](https://www-ncbi-nlm-nih-gov.gate2.inist.fr/pubmed/?term=Bahrami%20MH%5BAuthor%5D&cauthor=true&cauthor_uid=26502966), [Shahraeeni S](https://www-ncbi-nlm-nih-gov.gate2.inist.fr/pubmed/?term=Shahraeeni%20S%5BAuthor%5D&cauthor=true&cauthor_uid=26502966), [Raeissadat SA](https://www-ncbi-nlm-nih-gov.gate2.inist.fr/pubmed/?term=Raeissadat%20SA%5BAuthor%5D&cauthor=true&cauthor_uid=26502966). Comparison between the effects of progesterone versus corticosteroid local injections in mild and moderate carpal tunnel syndrome: a randomized clinical trial. [BMC Musculoskelet Disord.](https://www-ncbi-nlm-nih-gov.gate2.inist.fr/pubmed/?term=26502966) 2015 Oct 26;16:322

11. [Bangure D](https://www-ncbi-nlm-nih-gov.gate2.inist.fr/pubmed/?term=Bangure%20D%5BAuthor%5D&cauthor=true&cauthor_uid=25885862), [Chirundu D](https://www-ncbi-nlm-nih-gov.gate2.inist.fr/pubmed/?term=Chirundu%20D%5BAuthor%5D&cauthor=true&cauthor_uid=25885862), [Gombe N](https://www-ncbi-nlm-nih-gov.gate2.inist.fr/pubmed/?term=Gombe%20N%5BAuthor%5D&cauthor=true&cauthor_uid=25885862), et al. Effectiveness of short message services reminder on childhood immunizationprogramme in Kadoma, Zimbabwe - a randomized controlled trial, 2013. [BMC Public Health.](https://www-ncbi-nlm-nih-gov.gate2.inist.fr/pubmed/?term=Effectiveness+of+Short+Message+Services+Reminder+on+Childhood+Immunization+Programme+in+Kadoma%2C+Zimbabwe) 2015 Feb 12;15:137

12. [Berdah SV](https://www-ncbi-nlm-nih-gov.gate2.inist.fr/pubmed/?term=Berdah%20SV%5BAuthor%5D&cauthor=true&cauthor_uid=25348087), [Mariette C](https://www-ncbi-nlm-nih-gov.gate2.inist.fr/pubmed/?term=Mariette%20C%5BAuthor%5D&cauthor=true&cauthor_uid=25348087), [Denet C](https://www-ncbi-nlm-nih-gov.gate2.inist.fr/pubmed/?term=Denet%20C%5BAuthor%5D&cauthor=true&cauthor_uid=25348087), et al.A multicentre, randomised, controlled trial to assess the safety, ease of use, and reliability of hyaluronic acid/carboxymethylcellulose powder adhesion barrier versus no barrier in colorectal laparoscopic surgery. [Trials.](https://www-ncbi-nlm-nih-gov.gate2.inist.fr/pubmed/?term=A+multicentre%2C+randomised%2C+controlled+trial+to+assess+the+safety%2C+ease+of+use%2C+and+reliability+of+hyaluronic+acid%2Fcarboxymethylcellulose+powder+adhesion+barrier+versus+no+barrier+in+colorectal+laparoscopic+surgery) 2014 Oct 27;15:413

13. [Bernstein SL](https://www-ncbi-nlm-nih-gov.gate2.inist.fr/pubmed/?term=Bernstein%20SL%5BAuthor%5D&cauthor=true&cauthor_uid=25920384), [D'Onofrio G](https://www-ncbi-nlm-nih-gov.gate2.inist.fr/pubmed/?term=D%27Onofrio%20G%5BAuthor%5D&cauthor=true&cauthor_uid=25920384), [Rosner J](https://www-ncbi-nlm-nih-gov.gate2.inist.fr/pubmed/?term=Rosner%20J%5BAuthor%5D&cauthor=true&cauthor_uid=25920384), et al. Successful Tobacco Dependence Treatment in Low-Income Emergency Department Patients: A Randomized Trial. [Ann Emerg Med.](https://www-ncbi-nlm-nih-gov.gate2.inist.fr/pubmed/?term=25920384) 2015 Aug;66(2):140-7

14. [Biering-Sørensen S](https://www.ncbi.nlm.nih.gov/pubmed/?term=Biering-S%C3%B8rensen%20S%5BAuthor%5D&cauthor=true&cauthor_uid=26416147), [Andersen A](https://www.ncbi.nlm.nih.gov/pubmed/?term=Andersen%20A%5BAuthor%5D&cauthor=true&cauthor_uid=26416147), [Ravn H](https://www.ncbi.nlm.nih.gov/pubmed/?term=Ravn%20H%5BAuthor%5D&cauthor=true&cauthor_uid=26416147), et al. Early BCG vaccine to low-birth-weight infants and the effects on growth in the first year of life: a randomised controlled trial. [BMC Pediatr.](https://www.ncbi.nlm.nih.gov/pubmed/?term=26416147) 2015 Sep 28;15:137.

15. [Björling G](https://www-ncbi-nlm-nih-gov.gate2.inist.fr/pubmed/?term=Bj%C3%B6rling%20G%5BAuthor%5D&cauthor=true&cauthor_uid=26625869), [Johansson D](https://www-ncbi-nlm-nih-gov.gate2.inist.fr/pubmed/?term=Johansson%20D%5BAuthor%5D&cauthor=true&cauthor_uid=26625869), [Bergström L](https://www-ncbi-nlm-nih-gov.gate2.inist.fr/pubmed/?term=Bergstr%C3%B6m%20L%5BAuthor%5D&cauthor=true&cauthor_uid=26625869), et al. Tolerability and performance of BIP endotracheal tubes with noble metal alloy coating--a randomized clinical evaluation study. [BMC Anesthesiol.](https://www-ncbi-nlm-nih-gov.gate2.inist.fr/pubmed/?term=26625869) 2015 Dec 1;15:174

16. [Borja-Tabora CF](https://www.ncbi.nlm.nih.gov/pubmed/?term=Borja-Tabora%20CF%5BAuthor%5D&cauthor=true&cauthor_uid=26437712), [Montalban C](https://www.ncbi.nlm.nih.gov/pubmed/?term=Montalban%20C%5BAuthor%5D&cauthor=true&cauthor_uid=26437712), [Memish ZA](https://www.ncbi.nlm.nih.gov/pubmed/?term=Memish%20ZA%5BAuthor%5D&cauthor=true&cauthor_uid=26437712), et al. Long-term immunogenicity and safety after a single dose of the quadrivalent meningococcal serogroups A, C, W, and Y tetanus toxoid conjugate vaccine in adolescents and adults: 5-year follow-up of an open, randomized trial. [BMC Infect Dis.](https://www.ncbi.nlm.nih.gov/pubmed/?term=26437712%5Buid%5D) 2015 Oct 6;15:409.

17. [Buchanan H](https://www-ncbi-nlm-nih-gov.gate2.inist.fr/pubmed/?term=Buchanan%20H%5BAuthor%5D&cauthor=true&cauthor_uid=24916176), [Siegfried N](https://www-ncbi-nlm-nih-gov.gate2.inist.fr/pubmed/?term=Siegfried%20N%5BAuthor%5D&cauthor=true&cauthor_uid=24916176), [Jelsma J](https://www-ncbi-nlm-nih-gov.gate2.inist.fr/pubmed/?term=Jelsma%20J%5BAuthor%5D&cauthor=true&cauthor_uid=24916176), [et al](https://www-ncbi-nlm-nih-gov.gate2.inist.fr/pubmed/?term=Lombard%20C%5BAuthor%5D&cauthor=true&cauthor_uid=24916176). Comparison of an interactive with a didactic educational intervention for improving the evidence-based practice knowledge of occupational therapistsin the public health sector in South Africa: a randomised controlled trial. [Trials.](https://www-ncbi-nlm-nih-gov.gate2.inist.fr/pubmed/?term=Improving+the+Evidence+Based+Practice+of+Occupational+Therapists+in+the+Public+Health+Sector+in+South+Africa+through+an+Interactive+Educational+Intervention%3A+a+randomised+controlled+trial) 2014 Jun 10;15:216

18. [Buhse S](https://www-ncbi-nlm-nih-gov.gate2.inist.fr/pubmed/?term=Buhse%20S%5BAuthor%5D&cauthor=true&cauthor_uid=26567256), [Mühlhauser I](https://www-ncbi-nlm-nih-gov.gate2.inist.fr/pubmed/?term=M%C3%BChlhauser%20I%5BAuthor%5D&cauthor=true&cauthor_uid=26567256), [Heller T](https://www-ncbi-nlm-nih-gov.gate2.inist.fr/pubmed/?term=Heller%20T%5BAuthor%5D&cauthor=true&cauthor_uid=26567256), et al. Informed shared decision-making programme on the prevention of myocardial infarction in type 2 diabetes: a randomised controlled trial. [BMJ Open.](https://www-ncbi-nlm-nih-gov.gate2.inist.fr/pubmed/?term=26567256) 2015 Nov 13;5(11):e009116

19. [Carvalho RF](https://www-ncbi-nlm-nih-gov.gate2.inist.fr/pubmed/?term=Carvalho%20RF%5BAuthor%5D&cauthor=true&cauthor_uid=26077768), [Huguenin GV](https://www-ncbi-nlm-nih-gov.gate2.inist.fr/pubmed/?term=Huguenin%20GV%5BAuthor%5D&cauthor=true&cauthor_uid=26077768), [Luiz RR](https://www-ncbi-nlm-nih-gov.gate2.inist.fr/pubmed/?term=Luiz%20RR%5BAuthor%5D&cauthor=true&cauthor_uid=26077768), et al. Intake of partially defatted Brazil nut flour reduces serum cholesterol in hypercholesterolemic patients--a randomized controlled trial. [Nutr J.](https://www-ncbi-nlm-nih-gov.gate2.inist.fr/pubmed/?term=26077768) 2015 Jun 16;14:59

20. [Chang AK](https://www.ncbi.nlm.nih.gov/pubmed/?term=Chang%20AK%5BAuthor%5D&cauthor=true&cauthor_uid=23694801), [Bijur PE](https://www.ncbi.nlm.nih.gov/pubmed/?term=Bijur%20PE%5BAuthor%5D&cauthor=true&cauthor_uid=23694801), [Lupow JB](https://www.ncbi.nlm.nih.gov/pubmed/?term=Lupow%20JB%5BAuthor%5D&cauthor=true&cauthor_uid=23694801), et al. Randomized clinical trial of the 2 mg hydromorphone bolus protocol versus the "1+1" hydromorphone titration protocol in treatment of acute, severe pain in the first hour of emergency department presentation. [Ann Emerg Med.](https://www.ncbi.nlm.nih.gov/pubmed/?term=23694801) 2013 Oct;62(4):304-10

21. [Chinnock B](https://www-ncbi-nlm-nih-gov.gate2.inist.fr/pubmed/?term=Chinnock%20B%5BAuthor%5D&cauthor=true&cauthor_uid=26416494), [Hendey GW](https://www-ncbi-nlm-nih-gov.gate2.inist.fr/pubmed/?term=Hendey%20GW%5BAuthor%5D&cauthor=true&cauthor_uid=26416494). Irrigation of Cutaneous Abscesses Does Not Improve Treatment Success. [Ann Emerg Med.](https://www-ncbi-nlm-nih-gov.gate2.inist.fr/pubmed/?term=26416494%22) 2016 Mar;67(3):379-83

22. [Choi YJ](https://www-ncbi-nlm-nih-gov.gate2.inist.fr/pubmed/?term=Choi%20YJ%5BAuthor%5D&cauthor=true&cauthor_uid=26493267), [Lee DH](https://www-ncbi-nlm-nih-gov.gate2.inist.fr/pubmed/?term=Lee%20DH%5BAuthor%5D&cauthor=true&cauthor_uid=26493267), [Choi CM](https://www-ncbi-nlm-nih-gov.gate2.inist.fr/pubmed/?term=Choi%20CM%5BAuthor%5D&cauthor=true&cauthor_uid=26493267), et al. Randomized phase II study of paclitaxel/carboplatin intercalated with gefitinib compared to paclitaxel/carboplatin alone for chemotherapy-naïve non-small cell lung cancer in a clinically selected population excluding patients with non-smoking adenocarcinoma or mutated EGFR. [BMC Cancer.](https://www-ncbi-nlm-nih-gov.gate2.inist.fr/pubmed/?term=26493267) 2015 Oct 22;15:763

23. [Cohen SP](https://www-ncbi-nlm-nih-gov.gate2.inist.fr/pubmed/?term=Cohen%20SP%5BAuthor%5D&cauthor=true&cauthor_uid=25883095), [Hanling S](https://www-ncbi-nlm-nih-gov.gate2.inist.fr/pubmed/?term=Hanling%20S%5BAuthor%5D&cauthor=true&cauthor_uid=25883095), [Bicket MC](https://www-ncbi-nlm-nih-gov.gate2.inist.fr/pubmed/?term=Bicket%20MC%5BAuthor%5D&cauthor=true&cauthor_uid=25883095), et al. Epidural steroid injections compared with gabapentin for lumbosacral radicular pain: multicenter randomized double blind comparative efficacy study. [BMJ.](https://www-ncbi-nlm-nih-gov.gate2.inist.fr/pubmed/?term=25883095) 2015 Apr 16;350:h1748

24. [Currie S](https://www-ncbi-nlm-nih-gov.gate2.inist.fr/pubmed/?term=Currie%20S%5BAuthor%5D&cauthor=true&cauthor_uid=26686681), [Sinclair M](https://www-ncbi-nlm-nih-gov.gate2.inist.fr/pubmed/?term=Sinclair%20M%5BAuthor%5D&cauthor=true&cauthor_uid=26686681), [Liddle DS](https://www-ncbi-nlm-nih-gov.gate2.inist.fr/pubmed/?term=Liddle%20DS%5BAuthor%5D&cauthor=true&cauthor_uid=26686681), et al. Application of objective physical activity measurement in an antenatal physical activity consultation intervention: a randomised controlled trial. [BMC Public Health.](https://www-ncbi-nlm-nih-gov.gate2.inist.fr/pubmed/?term=26686681) 2015 Dec 18;15:1259

25. [Dart RC](https://www-ncbi-nlm-nih-gov.gate2.inist.fr/pubmed/?term=Dart%20RC%5BAuthor%5D&cauthor=true&cauthor_uid=23380292), [Bogdan G](https://www-ncbi-nlm-nih-gov.gate2.inist.fr/pubmed/?term=Bogdan%20G%5BAuthor%5D&cauthor=true&cauthor_uid=23380292), [Heard K](https://www-ncbi-nlm-nih-gov.gate2.inist.fr/pubmed/?term=Heard%20K%5BAuthor%5D&cauthor=true&cauthor_uid=23380292), et al. A randomized, double-blind, placebo-controlled trial of a highly purified equineF(ab)2 antibody black widow spider antivenom. [Ann Emerg Med.](https://www-ncbi-nlm-nih-gov.gate2.inist.fr/pubmed/?term=A+Randomized%2C+Double-Blind%2C+Placebo-Controlled+Trial+of+aHighly+Purified+Equine+F(ab)2+Antibody+Black+WidowSpider+Antivenom) 2013 Apr;61(4):458-67

26. [Drozdov D](https://www-ncbi-nlm-nih-gov.gate2.inist.fr/pubmed/?term=Drozdov%20D%5BAuthor%5D&cauthor=true&cauthor_uid=25934044), [Schwarz S](https://www-ncbi-nlm-nih-gov.gate2.inist.fr/pubmed/?term=Schwarz%20S%5BAuthor%5D&cauthor=true&cauthor_uid=25934044), [Kutz A](https://www-ncbi-nlm-nih-gov.gate2.inist.fr/pubmed/?term=Kutz%20A%5BAuthor%5D&cauthor=true&cauthor_uid=25934044), et al. Procalcitonin and pyuria-based algorithm reduces antibiotic use in urinary tract infections: a randomized controlled trial. [BMC Med.](https://www-ncbi-nlm-nih-gov.gate2.inist.fr/pubmed/?term=25934044%22) 2015 May 1;13:104

27. [El-Khoury F](https://www-ncbi-nlm-nih-gov.gate2.inist.fr/pubmed/?term=El-Khoury%20F%5BAuthor%5D&cauthor=true&cauthor_uid=26201510), [Cassou B](https://www-ncbi-nlm-nih-gov.gate2.inist.fr/pubmed/?term=Cassou%20B%5BAuthor%5D&cauthor=true&cauthor_uid=26201510), [Latouche A](https://www-ncbi-nlm-nih-gov.gate2.inist.fr/pubmed/?term=Latouche%20A%5BAuthor%5D&cauthor=true&cauthor_uid=26201510), et al. Effectiveness of two year balance training programme on prevention of fall induced injuries in at risk women aged 75-85 living in community: Ossébo randomised controlled trial. [BMJ.](https://www-ncbi-nlm-nih-gov.gate2.inist.fr/pubmed/?term=26201510) 2015 Jul 22;351:h3830

28. [Enander J](https://www-ncbi-nlm-nih-gov.gate2.inist.fr/pubmed/?term=Enander%20J%5BAuthor%5D&cauthor=true&cauthor_uid=26837684), [Andersson E](https://www-ncbi-nlm-nih-gov.gate2.inist.fr/pubmed/?term=Andersson%20E%5BAuthor%5D&cauthor=true&cauthor_uid=26837684), [Mataix-Cols D](https://www-ncbi-nlm-nih-gov.gate2.inist.fr/pubmed/?term=Mataix-Cols%20D%5BAuthor%5D&cauthor=true&cauthor_uid=26837684), et al. Therapist guided internet based cognitive behavioural therapy for body dysmorphic disorder: single blind randomised controlled trial. [BMJ.](https://www-ncbi-nlm-nih-gov.gate2.inist.fr/pubmed/?term=26837684) 2016 Feb 2;352:i241

29. [Feczko P](https://www-ncbi-nlm-nih-gov.gate2.inist.fr/pubmed/?term=Feczko%20P%5BAuthor%5D&cauthor=true&cauthor_uid=26762175), [Engelmann L](https://www-ncbi-nlm-nih-gov.gate2.inist.fr/pubmed/?term=Engelmann%20L%5BAuthor%5D&cauthor=true&cauthor_uid=26762175), [Arts JJ](https://www-ncbi-nlm-nih-gov.gate2.inist.fr/pubmed/?term=Arts%20JJ%5BAuthor%5D&cauthor=true&cauthor_uid=26762175), et al. Computer-assisted total knee arthroplasty using mini midvastus or medial parapatellar approach technique: A prospective, randomized, international multicentre trial. [BMC Musculoskelet Disord.](https://www-ncbi-nlm-nih-gov.gate2.inist.fr/pubmed/?term=26762175) 2016 Jan 13;17:19

30. [Firouzi S](https://www-ncbi-nlm-nih-gov.gate2.inist.fr/pubmed/?term=Firouzi%20S%5BAuthor%5D&cauthor=true&cauthor_uid=26654906), [Mohd-Yusof BN](https://www-ncbi-nlm-nih-gov.gate2.inist.fr/pubmed/?term=Mohd-Yusof%20BN%5BAuthor%5D&cauthor=true&cauthor_uid=26654906), [Majid HA](https://www-ncbi-nlm-nih-gov.gate2.inist.fr/pubmed/?term=Majid%20HA%5BAuthor%5D&cauthor=true&cauthor_uid=26654906), et al. Effect of microbial cell preparation on renal profile and liver function among type 2 diabetics: a randomized controlled trial. [BMC Complement Altern Med.](https://www-ncbi-nlm-nih-gov.gate2.inist.fr/pubmed/?term=26654906) 2015 Dec 12;15:433

31. [Fox MJ](https://www-ncbi-nlm-nih-gov.gate2.inist.fr/pubmed/?term=Fox%20MJ%5BAuthor%5D&cauthor=true&cauthor_uid=25588782), [Ahuja KD](https://www-ncbi-nlm-nih-gov.gate2.inist.fr/pubmed/?term=Ahuja%20KD%5BAuthor%5D&cauthor=true&cauthor_uid=25588782), [Robertson IK](https://www-ncbi-nlm-nih-gov.gate2.inist.fr/pubmed/?term=Robertson%20IK%5BAuthor%5D&cauthor=true&cauthor_uid=25588782), et al. Can probiotic yogurt prevent diarrhoea in children on antibiotics? A double-blind, randomised, placebo-controlled study. [BMJ Open.](https://www-ncbi-nlm-nih-gov.gate2.inist.fr/pubmed/?term=25588782) 2015 Jan 14;5(1):e006474

32. [Fraval A](https://www-ncbi-nlm-nih-gov.gate2.inist.fr/pubmed/?term=Fraval%20A%5BAuthor%5D&cauthor=true&cauthor_uid=25885962), [Chandrananth J](https://www-ncbi-nlm-nih-gov.gate2.inist.fr/pubmed/?term=Chandrananth%20J%5BAuthor%5D&cauthor=true&cauthor_uid=25885962), [Chong YM](https://www-ncbi-nlm-nih-gov.gate2.inist.fr/pubmed/?term=Chong%20YM%5BAuthor%5D&cauthor=true&cauthor_uid=25885962), et al. Internet based patient education improves informed consent for electiveorthopaedic surgery: a randomized controlled trial. [BMC Musculoskelet Disord.](https://www-ncbi-nlm-nih-gov.gate2.inist.fr/pubmed/25885962) 2015 Feb 7;16:14

33. [Friedman BW](https://www-ncbi-nlm-nih-gov.gate2.inist.fr/pubmed/?term=Friedman%20BW%5BAuthor%5D&cauthor=true&cauthor_uid=26320523), [Cabral L](https://www-ncbi-nlm-nih-gov.gate2.inist.fr/pubmed/?term=Cabral%20L%5BAuthor%5D&cauthor=true&cauthor_uid=26320523), [Adewunmi V](https://www-ncbi-nlm-nih-gov.gate2.inist.fr/pubmed/?term=Adewunmi%20V%5BAuthor%5D&cauthor=true&cauthor_uid=26320523),  et al. Diphenhydramine as Adjuvant Therapy for Acute Migraine: An Emergency Department-Based Randomized Clinical Trial. [Ann Emerg Med.](https://www-ncbi-nlm-nih-gov.gate2.inist.fr/pubmed/?term=26320523) 2016 Jan;67(1):32-39.e3

34. [Furyk JS](https://www-ncbi-nlm-nih-gov.gate2.inist.fr/pubmed/?term=Furyk%20JS%5BAuthor%5D&cauthor=true&cauthor_uid=26194935), [Chu K](https://www-ncbi-nlm-nih-gov.gate2.inist.fr/pubmed/?term=Chu%20K%5BAuthor%5D&cauthor=true&cauthor_uid=26194935), [Banks C](https://www-ncbi-nlm-nih-gov.gate2.inist.fr/pubmed/?term=Banks%20C%5BAuthor%5D&cauthor=true&cauthor_uid=26194935), et al. Distal Ureteric Stones and Tamsulosin: A Double-Blind, Placebo-Controlled, Randomized, Multicenter Trial. [Ann Emerg Med.](https://www-ncbi-nlm-nih-gov.gate2.inist.fr/pubmed/?term=26194935) 2016 Jan;67(1):86-95.e2

35. [Gágyor I](https://www-ncbi-nlm-nih-gov.gate2.inist.fr/pubmed/?term=G%C3%A1gyor%20I%5BAuthor%5D&cauthor=true&cauthor_uid=26698878), [Bleidorn J](https://www-ncbi-nlm-nih-gov.gate2.inist.fr/pubmed/?term=Bleidorn%20J%5BAuthor%5D&cauthor=true&cauthor_uid=26698878), [Kochen MM](https://www-ncbi-nlm-nih-gov.gate2.inist.fr/pubmed/?term=Kochen%20MM%5BAuthor%5D&cauthor=true&cauthor_uid=26698878), et al. Ibuprofen versus fosfomycin for uncomplicated urinary tract infection in women: randomised controlled trial. [BMJ.](https://www-ncbi-nlm-nih-gov.gate2.inist.fr/pubmed/?term=26698878) 2015 Dec 23;351:h6544

36. [Gao F](https://www-ncbi-nlm-nih-gov.gate2.inist.fr/pubmed/?term=Gao%20F%5BAuthor%5D&cauthor=true&cauthor_uid=26637992), [Sun W](https://www-ncbi-nlm-nih-gov.gate2.inist.fr/pubmed/?term=Sun%20W%5BAuthor%5D&cauthor=true&cauthor_uid=26637992), [Li Z](https://www-ncbi-nlm-nih-gov.gate2.inist.fr/pubmed/?term=Li%20Z%5BAuthor%5D&cauthor=true&cauthor_uid=26637992), et al. Extracorporeal shock wave therapy in the treatment of primary bone marrow edema syndrome of the knee: a prospective randomised controlled study. [BMC Musculoskelet Disord.](https://www-ncbi-nlm-nih-gov.gate2.inist.fr/pubmed/?term=26637992%5Buid%5D) 2015 Dec 5;16:379

37. [García-Padilla S](https://www-ncbi-nlm-nih-gov.gate2.inist.fr/pubmed/?term=Garc%C3%ADa-Padilla%20S%5BAuthor%5D&cauthor=true&cauthor_uid=25963758), [Duarte-Vázquez MA](https://www-ncbi-nlm-nih-gov.gate2.inist.fr/pubmed/?term=Duarte-V%C3%A1zquez%20MA%5BAuthor%5D&cauthor=true&cauthor_uid=25963758), [Gonzalez-Romero KE](https://www-ncbi-nlm-nih-gov.gate2.inist.fr/pubmed/?term=Gonzalez-Romero%20KE%5BAuthor%5D&cauthor=true&cauthor_uid=25963758), et al. Effectiveness of intra-articular injections of sodium bicarbonate and calcium gluconate in the treatment of osteoarthritis of the knee: a randomized double-blind clinical trial. [BMC Musculoskelet Disord.](https://www-ncbi-nlm-nih-gov.gate2.inist.fr/pubmed/?term=25963758) 2015 May 13;16:114

38. [Geukers VG](https://www-ncbi-nlm-nih-gov.gate2.inist.fr/pubmed/?term=Geukers%20VG%5BAuthor%5D&cauthor=true&cauthor_uid=26215396), [Dijsselhof ME](https://www-ncbi-nlm-nih-gov.gate2.inist.fr/pubmed/?term=Dijsselhof%20ME%5BAuthor%5D&cauthor=true&cauthor_uid=26215396), [Jansen NJ](https://www-ncbi-nlm-nih-gov.gate2.inist.fr/pubmed/?term=Jansen%20NJ%5BAuthor%5D&cauthor=true&cauthor_uid=26215396), et al. The effect of short-term high versus normal protein intake on whole-body protein synthesis and balance in children following cardiac surgery: a randomized double-blind controlled clinical trial. [Nutr J.](https://www-ncbi-nlm-nih-gov.gate2.inist.fr/pubmed/?term=26215396) 2015 Jul 28;14:72

39. [Gill TM](https://www-ncbi-nlm-nih-gov.gate2.inist.fr/pubmed/?term=Gill%20TM%5BAuthor%5D&cauthor=true&cauthor_uid=26842425), [Pahor M](https://www-ncbi-nlm-nih-gov.gate2.inist.fr/pubmed/?term=Pahor%20M%5BAuthor%5D&cauthor=true&cauthor_uid=26842425), [Guralnik JM](https://www-ncbi-nlm-nih-gov.gate2.inist.fr/pubmed/?term=Guralnik%20JM%5BAuthor%5D&cauthor=true&cauthor_uid=26842425), et al. Effect of structured physical activity on prevention of serious fall injuries in adults aged 70-89: randomized clinical trial (LIFE Study). [BMJ.](https://www-ncbi-nlm-nih-gov.gate2.inist.fr/pubmed/?term=26842425) 2016 Feb 3;352:i245

40. [Graudins A](https://www-ncbi-nlm-nih-gov.gate2.inist.fr/pubmed/?term=Graudins%20A%5BAuthor%5D&cauthor=true&cauthor_uid=25447557), [Meek R](https://www-ncbi-nlm-nih-gov.gate2.inist.fr/pubmed/?term=Meek%20R%5BAuthor%5D&cauthor=true&cauthor_uid=25447557), [Egerton-Warburton D](https://www-ncbi-nlm-nih-gov.gate2.inist.fr/pubmed/?term=Egerton-Warburton%20D%5BAuthor%5D&cauthor=true&cauthor_uid=25447557), et al. The PICHFORK (Pain in Children Fentanyl or Ketamine) trial: a randomized controlled trial comparing intranasal ketamine and fentanyl for the relief of moderate to severe pain in children with limb injuries. [Ann Emerg Med.](https://www-ncbi-nlm-nih-gov.gate2.inist.fr/pubmed/?term=25447557) 2015 Mar;65(3):248-254.e1

41. [Hall WA](https://www-ncbi-nlm-nih-gov.gate2.inist.fr/pubmed/?term=Hall%20WA%5BAuthor%5D&cauthor=true&cauthor_uid=26567090), [Hutton E](https://www-ncbi-nlm-nih-gov.gate2.inist.fr/pubmed/?term=Hutton%20E%5BAuthor%5D&cauthor=true&cauthor_uid=26567090), [Brant RF](https://www-ncbi-nlm-nih-gov.gate2.inist.fr/pubmed/?term=Brant%20RF%5BAuthor%5D&cauthor=true&cauthor_uid=26567090), et al. A randomized controlled trial of an intervention for infants' behavioral sleep problems. [BMC Pediatr.](https://www-ncbi-nlm-nih-gov.gate2.inist.fr/pubmed/?term=26567090) 2015 Nov 13;15:181

42. [Hess EP](https://www-ncbi-nlm-nih-gov.gate2.inist.fr/pubmed/?term=Hess%20EP%5BAuthor%5D&cauthor=true&cauthor_uid=27919865), [Hollander JE](https://www-ncbi-nlm-nih-gov.gate2.inist.fr/pubmed/?term=Hollander%20JE%5BAuthor%5D&cauthor=true&cauthor_uid=27919865), [Schaffer JT](https://www-ncbi-nlm-nih-gov.gate2.inist.fr/pubmed/?term=Schaffer%20JT%5BAuthor%5D&cauthor=true&cauthor_uid=27919865), et al. Shared decision making in patients with low risk chest pain: prospective randomized pragmatic trial. [BMJ.](https://www-ncbi-nlm-nih-gov.gate2.inist.fr/pubmed/?term=Shared+decision+making+in+patients+with+low+risk+chest+pain%3A+prospective+randomized+pragmatic+trial) 2016 Dec 5;355:i6165

43. [Hopper SM](https://www-ncbi-nlm-nih-gov.gate2.inist.fr/pubmed/?term=Hopper%20SM%5BAuthor%5D&cauthor=true&cauthor_uid=24210368), [McCarthy M](https://www-ncbi-nlm-nih-gov.gate2.inist.fr/pubmed/?term=McCarthy%20M%5BAuthor%5D&cauthor=true&cauthor_uid=24210368), [Tancharoen C](https://www-ncbi-nlm-nih-gov.gate2.inist.fr/pubmed/?term=Tancharoen%20C%5BAuthor%5D&cauthor=true&cauthor_uid=24210368), et al. Topical lidocaine to improve oral intake in children with painful infectious mouth ulcers: a blinded, randomized, placebo-controlled trial. [Ann Emerg Med.](https://www-ncbi-nlm-nih-gov.gate2.inist.fr/pubmed/?term=24210368) 2014 Mar;63(3):292-9

44. [Husted GR](https://www-ncbi-nlm-nih-gov.gate2.inist.fr/pubmed/?term=Husted%20GR%5BAuthor%5D&cauthor=true&cauthor_uid=25118146), [Thorsteinsson B](https://www-ncbi-nlm-nih-gov.gate2.inist.fr/pubmed/?term=Thorsteinsson%20B%5BAuthor%5D&cauthor=true&cauthor_uid=25118146), [Esbensen BA](https://www-ncbi-nlm-nih-gov.gate2.inist.fr/pubmed/?term=Esbensen%20BA%5BAuthor%5D&cauthor=true&cauthor_uid=25118146), et al. Effect of guided self-determination youth intervention integrated into outpatient visits versus treatment as usual on glycemic control and life skills: a randomized clinical trial in adolescents with type 1 diabetes. [Trials.](https://www-ncbi-nlm-nih-gov.gate2.inist.fr/pubmed/?term=Effect+of+guided+self-determination+youth+intervention+integrated+into+outpatient+visits+versus+treatment+as+usual+on+glycemic+control+and+life+skills%3A+a+randomized+clinical+trial+in+adolescents+with+type+1+diabetes) 2014 Aug 12;15:321.

45. [Hwang W](https://www.ncbi.nlm.nih.gov/pubmed/?term=Hwang%20W%5BAuthor%5D&cauthor=true&cauthor_uid=25750586), [Lee J](https://www.ncbi.nlm.nih.gov/pubmed/?term=Lee%20J%5BAuthor%5D&cauthor=true&cauthor_uid=25750586), [Park J](https://www.ncbi.nlm.nih.gov/pubmed/?term=Park%20J%5BAuthor%5D&cauthor=true&cauthor_uid=25750586), et al. Dexmedetomidine versus remifentanil in postoperative pain control after spinal surgery: a randomized controlled study. [BMC Anesthesiol.](https://www.ncbi.nlm.nih.gov/pubmed/?term=25750586%5Buid%5D) 2015 Feb 24;15:21.

46. [Isbister GK](https://www-ncbi-nlm-nih-gov.gate2.inist.fr/pubmed/?term=Isbister%20GK%5BAuthor%5D&cauthor=true&cauthor_uid=24999282), [Page CB](https://www-ncbi-nlm-nih-gov.gate2.inist.fr/pubmed/?term=Page%20CB%5BAuthor%5D&cauthor=true&cauthor_uid=24999282), [Buckley NA](https://www-ncbi-nlm-nih-gov.gate2.inist.fr/pubmed/?term=Buckley%20NA%5BAuthor%5D&cauthor=true&cauthor_uid=24999282), et al. Randomized controlled trial of intravenous antivenom versus placebo for latrodectism: the second Redback Antivenom Evaluation (RAVE-II) study. [Ann Emerg Med.](https://www-ncbi-nlm-nih-gov.gate2.inist.fr/pubmed/24999282) 2014 Dec;64(6):620-8.e2

47. [Jang JY](https://www-ncbi-nlm-nih-gov.gate2.inist.fr/pubmed/?term=Jang%20JY%5BAuthor%5D&cauthor=true&cauthor_uid=22901564), [Shin SD](https://www-ncbi-nlm-nih-gov.gate2.inist.fr/pubmed/?term=Shin%20SD%5BAuthor%5D&cauthor=true&cauthor_uid=22901564), [Lee EJ](https://www-ncbi-nlm-nih-gov.gate2.inist.fr/pubmed/?term=Lee%20EJ%5BAuthor%5D&cauthor=true&cauthor_uid=22901564), [Park CB](https://www-ncbi-nlm-nih-gov.gate2.inist.fr/pubmed/?term=Park%20CB%5BAuthor%5D&cauthor=true&cauthor_uid=22901564), [Song KJ](https://www-ncbi-nlm-nih-gov.gate2.inist.fr/pubmed/?term=Song%20KJ%5BAuthor%5D&cauthor=true&cauthor_uid=22901564), [Singer AJ](https://www-ncbi-nlm-nih-gov.gate2.inist.fr/pubmed/?term=Singer%20AJ%5BAuthor%5D&cauthor=true&cauthor_uid=22901564). Use of a comprehensive metabolic panel point-of-care test to reduce length of stay in the emergency department: a randomized controlled trial. [Ann Emerg Med.](https://www-ncbi-nlm-nih-gov.gate2.inist.fr/pubmed/?term=22901564) 2013 Feb;61(2):145-51

48. [Johnson MJ](https://www-ncbi-nlm-nih-gov.gate2.inist.fr/pubmed/?term=Johnson%20MJ%5BAuthor%5D&cauthor=true&cauthor_uid=26345362), [Kanaan M](https://www-ncbi-nlm-nih-gov.gate2.inist.fr/pubmed/?term=Kanaan%20M%5BAuthor%5D&cauthor=true&cauthor_uid=26345362), [Richardson G](https://www-ncbi-nlm-nih-gov.gate2.inist.fr/pubmed/?term=Richardson%20G%5BAuthor%5D&cauthor=true&cauthor_uid=26345362), et al. A randomised controlled trial of three or one breathing technique training sessions for breathlessness in people with malignant lung disease. [BMC Med.](https://www-ncbi-nlm-nih-gov.gate2.inist.fr/pubmed/?term=26345362) 2015 Sep 7;13:213

49. [Jonker AA](https://www-ncbi-nlm-nih-gov.gate2.inist.fr/pubmed/?term=Jonker%20AA%5BAuthor%5D&cauthor=true&cauthor_uid=26275714), [Comijs HC](https://www-ncbi-nlm-nih-gov.gate2.inist.fr/pubmed/?term=Comijs%20HC%5BAuthor%5D&cauthor=true&cauthor_uid=26275714), [Knipscheer KC](https://www-ncbi-nlm-nih-gov.gate2.inist.fr/pubmed/?term=Knipscheer%20KC%5BAuthor%5D&cauthor=true&cauthor_uid=26275714), et al. Benefits for elders with vulnerable health from the Chronic Disease Self-management Program (CDSMP) at short and longer term. [BMC Geriatr.](https://www-ncbi-nlm-nih-gov.gate2.inist.fr/pubmed/?term=26275714%22) 2015 Aug 15;15:101

50. [Kangasniemi AM](https://www-ncbi-nlm-nih-gov.gate2.inist.fr/pubmed/?term=Kangasniemi%20AM%5BAuthor%5D&cauthor=true&cauthor_uid=25848812), [Lappalainen R](https://www-ncbi-nlm-nih-gov.gate2.inist.fr/pubmed/?term=Lappalainen%20R%5BAuthor%5D&cauthor=true&cauthor_uid=25848812), [Kankaanpää A](https://www-ncbi-nlm-nih-gov.gate2.inist.fr/pubmed/?term=Kankaanp%C3%A4%C3%A4%20A%5BAuthor%5D&cauthor=true&cauthor_uid=25848812), et al. Towards a physically more active lifestyle based on one's own values: the results of a randomized controlled trial among physically inactive adults. [BMC Public Health.](https://www-ncbi-nlm-nih-gov.gate2.inist.fr/pubmed/?term=25848812) 2015 Mar 18;15:260

51. [Kassai B](https://www-ncbi-nlm-nih-gov.gate2.inist.fr/pubmed/?term=Kassai%20B%5BAuthor%5D&cauthor=true&cauthor_uid=26350209), [Rabilloud M](https://www-ncbi-nlm-nih-gov.gate2.inist.fr/pubmed/?term=Rabilloud%20M%5BAuthor%5D&cauthor=true&cauthor_uid=26350209), [Bernoux D](https://www-ncbi-nlm-nih-gov.gate2.inist.fr/pubmed/?term=Bernoux%20D%5BAuthor%5D&cauthor=true&cauthor_uid=26350209), et al. Management of adolescents with very poorly controlled type 1 diabetes by nurses: a parallel group randomized controlled trial. [Trials.](https://www-ncbi-nlm-nih-gov.gate2.inist.fr/pubmed/?term=26350209) 2015 Sep 8;16:399

52. [Kaufman J](https://www-ncbi-nlm-nih-gov.gate2.inist.fr/pubmed/?term=Kaufman%20J%5BAuthor%5D&cauthor=true&cauthor_uid=28389435), [Fitzpatrick P](https://www-ncbi-nlm-nih-gov.gate2.inist.fr/pubmed/?term=Fitzpatrick%20P%5BAuthor%5D&cauthor=true&cauthor_uid=28389435), [Tosif S](https://www-ncbi-nlm-nih-gov.gate2.inist.fr/pubmed/?term=Tosif%20S%5BAuthor%5D&cauthor=true&cauthor_uid=28389435), et al. Faster clean catch urine collection (Quick-Wee method) from infants: randomised controlled trial. [BMJ.](https://www-ncbi-nlm-nih-gov.gate2.inist.fr/pubmed/?term=Faster+clean+catch+urine+collection+(Quick-Wee+method)+from+infants%3A+randomised+controlled+trial) 2017 Apr 7;357:j1341

53. [Kehlet M](https://www-ncbi-nlm-nih-gov.gate2.inist.fr/pubmed/?term=Kehlet%20M%5BAuthor%5D&cauthor=true&cauthor_uid=26438129), [Heeseman S](https://www-ncbi-nlm-nih-gov.gate2.inist.fr/pubmed/?term=Heeseman%20S%5BAuthor%5D&cauthor=true&cauthor_uid=26438129), [Tønnesen H](https://www-ncbi-nlm-nih-gov.gate2.inist.fr/pubmed/?term=T%C3%B8nnesen%20H%5BAuthor%5D&cauthor=true&cauthor_uid=26438129), [et al](https://www-ncbi-nlm-nih-gov.gate2.inist.fr/pubmed/?term=Schroeder%20TV%5BAuthor%5D&cauthor=true&cauthor_uid=26438129). Perioperative smoking cessation in vascular surgery: challenges with a randomized controlled trial. [Trials.](https://www-ncbi-nlm-nih-gov.gate2.inist.fr/pubmed/?term=26438129) 2015 Oct 5;16:441.

54. [Kenyon S](https://www-ncbi-nlm-nih-gov.gate2.inist.fr/pubmed/?term=Kenyon%20S%5BAuthor%5D&cauthor=true&cauthor_uid=26936901), [Jolly K](https://www-ncbi-nlm-nih-gov.gate2.inist.fr/pubmed/?term=Jolly%20K%5BAuthor%5D&cauthor=true&cauthor_uid=26936901), [Hemming K](https://www-ncbi-nlm-nih-gov.gate2.inist.fr/pubmed/?term=Hemming%20K%5BAuthor%5D&cauthor=true&cauthor_uid=26936901), et al. Lay support for pregnant women with social risk: a randomised controlled trial. [BMJ Open.](https://www-ncbi-nlm-nih-gov.gate2.inist.fr/pubmed/?term=26936901) 2016 Mar 2;6(3):e009203

55. [Khanal A](https://www-ncbi-nlm-nih-gov.gate2.inist.fr/pubmed/?term=Khanal%20A%5BAuthor%5D&cauthor=true&cauthor_uid=26357896), [Sharma A](https://www-ncbi-nlm-nih-gov.gate2.inist.fr/pubmed/?term=Sharma%20A%5BAuthor%5D&cauthor=true&cauthor_uid=26357896), [Basnet S](https://www-ncbi-nlm-nih-gov.gate2.inist.fr/pubmed/?term=Basnet%20S%5BAuthor%5D&cauthor=true&cauthor_uid=26357896), et al. Nebulised hypertonic saline (3%) among children with mild to moderately severe bronchiolitis--a double blind randomized controlled trial. [BMC Pediatr.](https://www-ncbi-nlm-nih-gov.gate2.inist.fr/pubmed/?term=26357896) 2015 Sep 10;15:115.

56. [Kinung'hi SM](https://www-ncbi-nlm-nih-gov.gate2.inist.fr/pubmed/?term=Kinung%27hi%20SM%5BAuthor%5D&cauthor=true&cauthor_uid=25887977), [Magnussen P](https://www-ncbi-nlm-nih-gov.gate2.inist.fr/pubmed/?term=Magnussen%20P%5BAuthor%5D&cauthor=true&cauthor_uid=25887977), [Kishamawe C](https://www-ncbi-nlm-nih-gov.gate2.inist.fr/pubmed/?term=Kishamawe%20C%5BAuthor%5D&cauthor=true&cauthor_uid=25887977), et al. The impact of anthelmintic treatment intervention on malaria infection and anaemia in school and preschool children in Magu district, Tanzania: an open label randomised intervention trial. [BMC Infect Dis.](https://www-ncbi-nlm-nih-gov.gate2.inist.fr/pubmed/?term=25887977) 2015 Mar 20;15:136

57. [Kuan WS](https://www-ncbi-nlm-nih-gov.gate2.inist.fr/pubmed/?term=Kuan%20WS%5BAuthor%5D&cauthor=true&cauthor_uid=26475246), [Ibrahim I](https://www-ncbi-nlm-nih-gov.gate2.inist.fr/pubmed/?term=Ibrahim%20I%5BAuthor%5D&cauthor=true&cauthor_uid=26475246), [Leong BS](https://www-ncbi-nlm-nih-gov.gate2.inist.fr/pubmed/?term=Leong%20BS%5BAuthor%5D&cauthor=true&cauthor_uid=26475246), et al. Emergency Department Management of Sepsis Patients: A Randomized, Goal-Oriented, Noninvasive Sepsis Trial. [Ann Emerg Med.](https://www-ncbi-nlm-nih-gov.gate2.inist.fr/pubmed/?term=26475246) 2016 Mar;67(3):367-378.e3

58. [Kumar R](https://www-ncbi-nlm-nih-gov.gate2.inist.fr/pubmed/?term=Kumar%20R%5BAuthor%5D&cauthor=true&cauthor_uid=26847071), [Basu A](https://www-ncbi-nlm-nih-gov.gate2.inist.fr/pubmed/?term=Basu%20A%5BAuthor%5D&cauthor=true&cauthor_uid=26847071), [Sinha S](https://www-ncbi-nlm-nih-gov.gate2.inist.fr/pubmed/?term=Sinha%20S%5BAuthor%5D&cauthor=true&cauthor_uid=26847071), et al. Role of oral Minocycline in acute encephalitis syndrome in India - a randomized controlled trial. [BMC Infect Dis.](https://www-ncbi-nlm-nih-gov.gate2.inist.fr/pubmed/?term=26847071) 2016 Feb 4;16:67

59. [Kurz I](https://www-ncbi-nlm-nih-gov.gate2.inist.fr/pubmed/?term=Kurz%20I%5BAuthor%5D&cauthor=true&cauthor_uid=26944706), [Gimmon Y](https://www-ncbi-nlm-nih-gov.gate2.inist.fr/pubmed/?term=Gimmon%20Y%5BAuthor%5D&cauthor=true&cauthor_uid=26944706), [Shapiro A](https://www-ncbi-nlm-nih-gov.gate2.inist.fr/pubmed/?term=Shapiro%20A%5BAuthor%5D&cauthor=true&cauthor_uid=26944706), et al. Unexpected perturbations training improves balance control and voluntary stepping times in older adults - a double blind randomized control trial. BMC geriatrics. 2016 Mar 4;16:58

60. [Kwon HJ](https://www-ncbi-nlm-nih-gov.gate2.inist.fr/pubmed/?term=Kwon%20HJ%5BAuthor%5D&cauthor=true&cauthor_uid=26037730), [Choi JY](https://www-ncbi-nlm-nih-gov.gate2.inist.fr/pubmed/?term=Choi%20JY%5BAuthor%5D&cauthor=true&cauthor_uid=26037730), [Lee MS](https://www-ncbi-nlm-nih-gov.gate2.inist.fr/pubmed/?term=Lee%20MS%5BAuthor%5D&cauthor=true&cauthor_uid=26037730), et al. Acupuncture for the sequelae of Bell's palsy: a randomized controlled trial. [Trials.](https://www-ncbi-nlm-nih-gov.gate2.inist.fr/pubmed/?term=26037730) 2015 Jun 3;16:246

61. [Lee SJ](https://www-ncbi-nlm-nih-gov.gate2.inist.fr/pubmed/?term=Lee%20SJ%5BAuthor%5D&cauthor=true&cauthor_uid=26468007), [Kim S](https://www-ncbi-nlm-nih-gov.gate2.inist.fr/pubmed/?term=Kim%20S%5BAuthor%5D&cauthor=true&cauthor_uid=26468007), [Kim M](https://www-ncbi-nlm-nih-gov.gate2.inist.fr/pubmed/?term=Kim%20M%5BAuthor%5D&cauthor=true&cauthor_uid=26468007), et al. Capecitabine in combination with either cisplatin or weekly paclitaxel as a first-line treatment for metastatic esophageal squamous cell carcinoma: a randomized phase II study. [BMC Cancer.](https://www-ncbi-nlm-nih-gov.gate2.inist.fr/pubmed/?term=26468007) 2015 Oct 14;15:693

62. [Levy JA](https://www-ncbi-nlm-nih-gov.gate2.inist.fr/pubmed/?term=Levy%20JA%5BAuthor%5D&cauthor=true&cauthor_uid=22959318), [Bachur RG](https://www-ncbi-nlm-nih-gov.gate2.inist.fr/pubmed/?term=Bachur%20RG%5BAuthor%5D&cauthor=true&cauthor_uid=22959318), [Monuteaux MC](https://www-ncbi-nlm-nih-gov.gate2.inist.fr/pubmed/?term=Monuteaux%20MC%5BAuthor%5D&cauthor=true&cauthor_uid=22959318), et al. Intravenous dextrose for children with gastroenteritis and dehydration: a double-blind randomized controlled trial. [Ann Emerg Med.](https://www-ncbi-nlm-nih-gov.gate2.inist.fr/pubmed/?term=22959318) 2013 Mar;61(3):281-8

63. [Li J](https://www-ncbi-nlm-nih-gov.gate2.inist.fr/pubmed/?term=Li%20J%5BAuthor%5D&cauthor=true&cauthor_uid=26503370), [Li J](https://www-ncbi-nlm-nih-gov.gate2.inist.fr/pubmed/?term=Li%20J%5BAuthor%5D&cauthor=true&cauthor_uid=26503370), [Thornicroft G](https://www-ncbi-nlm-nih-gov.gate2.inist.fr/pubmed/?term=Thornicroft%20G%5BAuthor%5D&cauthor=true&cauthor_uid=26503370), et al. Training community mental health staff in Guangzhou, China: evaluation of the effect of a new training model. [BMC Psychiatry.](https://www-ncbi-nlm-nih-gov.gate2.inist.fr/pubmed/?term=26503370) 2015 Oct 26;15:263

64. [Li Q](https://www-ncbi-nlm-nih-gov.gate2.inist.fr/pubmed/?term=Li%20Q%5BAuthor%5D&cauthor=true&cauthor_uid=26445777), [Chen H](https://www-ncbi-nlm-nih-gov.gate2.inist.fr/pubmed/?term=Chen%20H%5BAuthor%5D&cauthor=true&cauthor_uid=26445777), [Hao JJ](https://www-ncbi-nlm-nih-gov.gate2.inist.fr/pubmed/?term=Hao%20JJ%5BAuthor%5D&cauthor=true&cauthor_uid=26445777), et al. Agreement of measured and calculated serum osmolality during the infusion of mannitol or hypertonic saline in patients after craniotomy: a prospective, double-blinded, randomised controlled trial. [BMC Anesthesiol.](https://www-ncbi-nlm-nih-gov.gate2.inist.fr/pubmed/?term=26445777) 2015 Oct 7;15:138

65. [Liu J](https://www-ncbi-nlm-nih-gov.gate2.inist.fr/pubmed/?term=Liu%20J%5BAuthor%5D&cauthor=true&cauthor_uid=25653009), [Chen P](https://www-ncbi-nlm-nih-gov.gate2.inist.fr/pubmed/?term=Chen%20P%5BAuthor%5D&cauthor=true&cauthor_uid=25653009), [Wang R](https://www-ncbi-nlm-nih-gov.gate2.inist.fr/pubmed/?term=Wang%20R%5BAuthor%5D&cauthor=true&cauthor_uid=25653009), et al. Effect of Tai Chi on mononuclear cell functions in patients with non-small cell lung cancer. [BMC Complement Altern Med.](https://www-ncbi-nlm-nih-gov.gate2.inist.fr/pubmed/?term=25653009) 2015 Feb 5;15:3

66. [Liu Z](https://www-ncbi-nlm-nih-gov.gate2.inist.fr/pubmed/?term=Liu%20Z%5BAuthor%5D&cauthor=true&cauthor_uid=25881090), [Li C](https://www-ncbi-nlm-nih-gov.gate2.inist.fr/pubmed/?term=Li%20C%5BAuthor%5D&cauthor=true&cauthor_uid=25881090), [Huang M](https://www-ncbi-nlm-nih-gov.gate2.inist.fr/pubmed/?term=Huang%20M%5BAuthor%5D&cauthor=true&cauthor_uid=25881090), et al. Positive regulatory effects of perioperative probiotic treatment on postoperative liver complications after colorectal liver metastases surgery: a double-center and double-blind randomized clinical trial. [BMC Gastroenterol.](https://www-ncbi-nlm-nih-gov.gate2.inist.fr/pubmed/?term=25881090) 2015 Mar 20;15:34

67. [Lin T](https://www-ncbi-nlm-nih-gov.gate2.inist.fr/pubmed/?term=Lin%20T%5BAuthor%5D&cauthor=true&cauthor_uid=26445425), [Liu J](https://www-ncbi-nlm-nih-gov.gate2.inist.fr/pubmed/?term=Liu%20J%5BAuthor%5D&cauthor=true&cauthor_uid=26445425), [Xiao B](https://www-ncbi-nlm-nih-gov.gate2.inist.fr/pubmed/?term=Xiao%20B%5BAuthor%5D&cauthor=true&cauthor_uid=26445425), et al. Comparison of the outcomes of cannulated screws vs. modified tension band wiring fixation techniques in the management of mildly displaced patellar fractures. [BMC Musculoskelet Disord.](https://www-ncbi-nlm-nih-gov.gate2.inist.fr/pubmed/?term=26445425) 2015 Oct 6;16:282

68. [Lind M](https://www-ncbi-nlm-nih-gov.gate2.inist.fr/pubmed/?term=Lind%20M%5BAuthor%5D&cauthor=true&cauthor_uid=26512041), [Hirsch IB](https://www-ncbi-nlm-nih-gov.gate2.inist.fr/pubmed/?term=Hirsch%20IB%5BAuthor%5D&cauthor=true&cauthor_uid=26512041), [Tuomilehto J](https://www-ncbi-nlm-nih-gov.gate2.inist.fr/pubmed/?term=Tuomilehto%20J%5BAuthor%5D&cauthor=true&cauthor_uid=26512041), et al. Liraglutide in people treated for type 2 diabetes with multiple daily insulin injections: randomised clinical trial (MDI Liraglutide trial). [BMJ.](https://www-ncbi-nlm-nih-gov.gate2.inist.fr/pubmed/?term=26512041) 2015 Oct 28;351:h5364

69. [Luangchosiri C](https://www-ncbi-nlm-nih-gov.gate2.inist.fr/pubmed/?term=Luangchosiri%20C%5BAuthor%5D&cauthor=true&cauthor_uid=26400476), [Thakkinstian A](https://www-ncbi-nlm-nih-gov.gate2.inist.fr/pubmed/?term=Thakkinstian%20A%5BAuthor%5D&cauthor=true&cauthor_uid=26400476), [Chitphuk S](https://www-ncbi-nlm-nih-gov.gate2.inist.fr/pubmed/?term=Chitphuk%20S%5BAuthor%5D&cauthor=true&cauthor_uid=26400476), et al. A double-blinded randomized controlled trial of silymarin for the prevention of antituberculosis drug-induced liver injury. [BMC Complement Altern Med.](https://www-ncbi-nlm-nih-gov.gate2.inist.fr/pubmed/?term=26400476) 2015 Sep 23;15:334

70. [Luedtke K](https://www-ncbi-nlm-nih-gov.gate2.inist.fr/pubmed/?term=Luedtke%20K%5BAuthor%5D&cauthor=true&cauthor_uid=25883244), [Rushton A](https://www-ncbi-nlm-nih-gov.gate2.inist.fr/pubmed/?term=Rushton%20A%5BAuthor%5D&cauthor=true&cauthor_uid=25883244), [Wright C](https://www-ncbi-nlm-nih-gov.gate2.inist.fr/pubmed/?term=Wright%20C%5BAuthor%5D&cauthor=true&cauthor_uid=25883244), et al. Effectiveness of transcranial direct current stimulation preceding cognitive behavioural management for chronic low back pain: sham controlled double blinded randomised controlled trial. [BMJ.](https://www-ncbi-nlm-nih-gov.gate2.inist.fr/pubmed/?term=25883244) 2015 Apr 16;350:h1640

71. [Markovic-Bozic J](https://www-ncbi-nlm-nih-gov.gate2.inist.fr/pubmed/?term=Markovic-Bozic%20J%5BAuthor%5D&cauthor=true&cauthor_uid=27001425), [Karpe B](https://www-ncbi-nlm-nih-gov.gate2.inist.fr/pubmed/?term=Karpe%20B%5BAuthor%5D&cauthor=true&cauthor_uid=27001425), [Potocnik I](https://www-ncbi-nlm-nih-gov.gate2.inist.fr/pubmed/?term=Potocnik%20I%5BAuthor%5D&cauthor=true&cauthor_uid=27001425), et al. Effect of propofol and sevoflurane on the inflammatory response of patients undergoing craniotomy. [BMC Anesthesiol.](https://www-ncbi-nlm-nih-gov.gate2.inist.fr/pubmed/?term=27001425%5Buid%5D) 2016 Mar 22;16:18

72. [Martins WR](https://www-ncbi-nlm-nih-gov.gate2.inist.fr/pubmed/?term=Martins%20WR%5BAuthor%5D&cauthor=true&cauthor_uid=26265075), [Safons MP](https://www-ncbi-nlm-nih-gov.gate2.inist.fr/pubmed/?term=Safons%20MP%5BAuthor%5D&cauthor=true&cauthor_uid=26265075), [Bottaro M](https://www-ncbi-nlm-nih-gov.gate2.inist.fr/pubmed/?term=Bottaro%20M%5BAuthor%5D&cauthor=true&cauthor_uid=26265075), et al. Effects of short term elastic resistance training on muscle mass and strength in untrained older adults: a randomized clinical trial. [BMC Geriatr.](https://www-ncbi-nlm-nih-gov.gate2.inist.fr/pubmed/?term=26265075%22) 2015 Aug 12;15:99

73. [McCann TV](https://www-ncbi-nlm-nih-gov.gate2.inist.fr/pubmed/?term=McCann%20TV%5BAuthor%5D&cauthor=true&cauthor_uid=26489756), [Songprakun W](https://www-ncbi-nlm-nih-gov.gate2.inist.fr/pubmed/?term=Songprakun%20W%5BAuthor%5D&cauthor=true&cauthor_uid=26489756), [Stephenson J](https://www-ncbi-nlm-nih-gov.gate2.inist.fr/pubmed/?term=Stephenson%20J%5BAuthor%5D&cauthor=true&cauthor_uid=26489756). Effectiveness of guided self-help in decreasing expressed emotion in family caregivers of people diagnosed with depression in Thailand: a randomised controlled trial. [BMC Psychiatry.](https://www-ncbi-nlm-nih-gov.gate2.inist.fr/pubmed/?term=26489756) 2015 Oct 21;15:258

74. [Mello MJ](https://www.ncbi.nlm.nih.gov/pubmed/?term=Mello%20MJ%5BAuthor%5D&cauthor=true&cauthor_uid=26585044), [Baird J](https://www.ncbi.nlm.nih.gov/pubmed/?term=Baird%20J%5BAuthor%5D&cauthor=true&cauthor_uid=26585044), [Lee C](https://www.ncbi.nlm.nih.gov/pubmed/?term=Lee%20C%5BAuthor%5D&cauthor=true&cauthor_uid=26585044), et al. A Randomized Controlled Trial of a Telephone Intervention for Alcohol Misuse With Injured Emergency Department Patients. [Ann Emerg Med.](https://www.ncbi.nlm.nih.gov/pubmed/?term=26585044%5Buid%5D) 2016 Feb;67(2):263-75.

75. [Mikor A](https://www-ncbi-nlm-nih-gov.gate2.inist.fr/pubmed/?term=Mikor%20A%5BAuthor%5D&cauthor=true&cauthor_uid=26041437), [Trásy D](https://www-ncbi-nlm-nih-gov.gate2.inist.fr/pubmed/?term=Tr%C3%A1sy%20D%5BAuthor%5D&cauthor=true&cauthor_uid=26041437), [Németh MF](https://www-ncbi-nlm-nih-gov.gate2.inist.fr/pubmed/?term=N%C3%A9meth%20MF%5BAuthor%5D&cauthor=true&cauthor_uid=26041437), et al. Continuous central venous oxygen saturation assisted intraoperative hemodynamic management during major abdominal surgery: a randomized, controlled trial. [BMC Anesthesiol.](https://www-ncbi-nlm-nih-gov.gate2.inist.fr/pubmed/?term=26041437%22) 2015 Jun 4;15:82

76. [Molinari N](https://www-ncbi-nlm-nih-gov.gate2.inist.fr/pubmed/?term=Molinari%20N%5BAuthor%5D&cauthor=true&cauthor_uid=25927404), [Abou-Badra M](https://www-ncbi-nlm-nih-gov.gate2.inist.fr/pubmed/?term=Abou-Badra%20M%5BAuthor%5D&cauthor=true&cauthor_uid=25927404), [Marin G](https://www-ncbi-nlm-nih-gov.gate2.inist.fr/pubmed/?term=Marin%20G%5BAuthor%5D&cauthor=true&cauthor_uid=25927404), et al. Is generalization of exhaled CO assessment in primary care helpful for early diagnosis of COPD? [BMC Pulm Med.](https://www-ncbi-nlm-nih-gov.gate2.inist.fr/pubmed/?term=25927404) 2015 Apr 28;15:44

77. [Montenegro ML](https://www-ncbi-nlm-nih-gov.gate2.inist.fr/pubmed/?term=Montenegro%20ML%5BAuthor%5D&cauthor=true&cauthor_uid=26628263), [Braz CA](https://www-ncbi-nlm-nih-gov.gate2.inist.fr/pubmed/?term=Braz%20CA%5BAuthor%5D&cauthor=true&cauthor_uid=26628263), [Rosa-e-Silva JC](https://www-ncbi-nlm-nih-gov.gate2.inist.fr/pubmed/?term=Rosa-e-Silva%20JC%5BAuthor%5D&cauthor=true&cauthor_uid=26628263), et al. Anaesthetic injection versus ischemic compression for the pain relief of abdominal wall trigger points in women with chronic pelvic pain. [BMC Anesthesiol.](https://www-ncbi-nlm-nih-gov.gate2.inist.fr/pubmed/?term=26628263) 2015 Dec 1;15:175

78. [Motov S](https://www-ncbi-nlm-nih-gov.gate2.inist.fr/pubmed/?term=Motov%20S%5BAuthor%5D&cauthor=true&cauthor_uid=25817884), [Rockoff B](https://www-ncbi-nlm-nih-gov.gate2.inist.fr/pubmed/?term=Rockoff%20B%5BAuthor%5D&cauthor=true&cauthor_uid=25817884), [Cohen V](https://www-ncbi-nlm-nih-gov.gate2.inist.fr/pubmed/?term=Cohen%20V%5BAuthor%5D&cauthor=true&cauthor_uid=25817884), et al. Intravenous Subdissociative-Dose Ketamine Versus Morphine for Analgesia in the Emergency Department: A Randomized Controlled Trial. [Ann Emerg Med.](https://www-ncbi-nlm-nih-gov.gate2.inist.fr/pubmed/?term=25817884) 2015 Sep;66(3):222-229.e1

79. [Nayeri F](https://www-ncbi-nlm-nih-gov.gate2.inist.fr/pubmed/?term=Nayeri%20F%5BAuthor%5D&cauthor=true&cauthor_uid=25976238), [Sheikh M](https://www-ncbi-nlm-nih-gov.gate2.inist.fr/pubmed/?term=Sheikh%20M%5BAuthor%5D&cauthor=true&cauthor_uid=25976238), [Kalani M](https://www-ncbi-nlm-nih-gov.gate2.inist.fr/pubmed/?term=Kalani%20M%5BAuthor%5D&cauthor=true&cauthor_uid=25976238), et al. Phenobarbital versus morphine in the management of neonatal abstinence syndrome, a randomized control trial. [BMC Pediatr.](https://www-ncbi-nlm-nih-gov.gate2.inist.fr/pubmed/?term=25976238) 2015 May 15;15:57

80. [Ng KP](https://www-ncbi-nlm-nih-gov.gate2.inist.fr/pubmed/?term=Ng%20KP%5BAuthor%5D&cauthor=true&cauthor_uid=26916697), [Jain P](https://www-ncbi-nlm-nih-gov.gate2.inist.fr/pubmed/?term=Jain%20P%5BAuthor%5D&cauthor=true&cauthor_uid=26916697), [Gill PS](https://www-ncbi-nlm-nih-gov.gate2.inist.fr/pubmed/?term=Gill%20PS%5BAuthor%5D&cauthor=true&cauthor_uid=26916697), et al. Results and lessons from the Spironolactone To Prevent Cardiovascular Events in Early Stage Chronic Kidney Disease (STOP-CKD) randomised controlled trial. [BMJ Open.](https://www-ncbi-nlm-nih-gov.gate2.inist.fr/pubmed/?term=26916697) 2016 Feb 25;6(2):e010519

81. [Ngo-Matip ME](https://www-ncbi-nlm-nih-gov.gate2.inist.fr/pubmed/?term=Ngo-Matip%20ME%5BAuthor%5D&cauthor=true&cauthor_uid=26195001), [Pieme CA](https://www-ncbi-nlm-nih-gov.gate2.inist.fr/pubmed/?term=Pieme%20CA%5BAuthor%5D&cauthor=true&cauthor_uid=26195001), [Azabji-Kenfack M](https://www-ncbi-nlm-nih-gov.gate2.inist.fr/pubmed/?term=Azabji-Kenfack%20M%5BAuthor%5D&cauthor=true&cauthor_uid=26195001), et al. Impact of daily supplementation of Spirulina platensis on the immune system of naïve HIV-1 patients in Cameroon: a 12-months single blind, randomized, multicenter trial. [Nutr J.](https://www-ncbi-nlm-nih-gov.gate2.inist.fr/pubmed/?term=26195001) 2015 Jul 21;14:70

82. [Noskova P](https://www-ncbi-nlm-nih-gov.gate2.inist.fr/pubmed/?term=Noskova%20P%5BAuthor%5D&cauthor=true&cauthor_uid=25821405), [Blaha J](https://www-ncbi-nlm-nih-gov.gate2.inist.fr/pubmed/?term=Blaha%20J%5BAuthor%5D&cauthor=true&cauthor_uid=25821405), [Bakhouche H](https://www-ncbi-nlm-nih-gov.gate2.inist.fr/pubmed/?term=Bakhouche%20H%5BAuthor%5D&cauthor=true&cauthor_uid=25821405), et al. Neonatal effect of remifentanil in general anaesthesia for caesarean section: a randomized trial. [BMC Anesthesiol.](https://www-ncbi-nlm-nih-gov.gate2.inist.fr/pubmed/?term=25821405) 2015 Mar 26;15:38

83. [Olsson SJ](https://www-ncbi-nlm-nih-gov.gate2.inist.fr/pubmed/?term=Olsson%20SJ%5BAuthor%5D&cauthor=true&cauthor_uid=26193882), [Börjesson M](https://www-ncbi-nlm-nih-gov.gate2.inist.fr/pubmed/?term=B%C3%B6rjesson%20M%5BAuthor%5D&cauthor=true&cauthor_uid=26193882), [Ekblom-Bak E](https://www-ncbi-nlm-nih-gov.gate2.inist.fr/pubmed/?term=Ekblom-Bak%20E%5BAuthor%5D&cauthor=true&cauthor_uid=26193882), et al. Effects of the Swedish physical activity on prescription model on health-related quality of life in overweight older adults: a randomised controlled trial. [BMC Public Health.](https://www-ncbi-nlm-nih-gov.gate2.inist.fr/pubmed/?term=26193882) 2015 Jul 21;15:687

84. [Palmer K](https://www-ncbi-nlm-nih-gov.gate2.inist.fr/pubmed/?term=Palmer%20K%5BAuthor%5D&cauthor=true&cauthor_uid=25935843), [Hebron C](https://www-ncbi-nlm-nih-gov.gate2.inist.fr/pubmed/?term=Hebron%20C%5BAuthor%5D&cauthor=true&cauthor_uid=25935843), [Williams JM](https://www-ncbi-nlm-nih-gov.gate2.inist.fr/pubmed/?term=Williams%20JM%5BAuthor%5D&cauthor=true&cauthor_uid=25935843). A randomised trial into the effect of an isolated hip abductor strengthening programme and a functional motor control programme on knee kinematics and hip muscle strength. [BMC Musculoskelet Disord.](https://www-ncbi-nlm-nih-gov.gate2.inist.fr/pubmed/?term=25935843) 2015 May 3;16:105

85. [Pan A](https://www-ncbi-nlm-nih-gov.gate2.inist.fr/pubmed/?term=Pan%20A%5BAuthor%5D&cauthor=true&cauthor_uid=25888092), [Alansari M](https://www-ncbi-nlm-nih-gov.gate2.inist.fr/pubmed/?term=Alansari%20M%5BAuthor%5D&cauthor=true&cauthor_uid=25888092), [Lubcke R](https://www-ncbi-nlm-nih-gov.gate2.inist.fr/pubmed/?term=Lubcke%20R%5BAuthor%5D&cauthor=true&cauthor_uid=25888092), et al. Use of pethidine for percutaneous liver biopsy - a randomised, placebo-controlled, double blind study. [BMC Gastroenterol.](https://www-ncbi-nlm-nih-gov.gate2.inist.fr/pubmed/?term=25888092) 2015 Mar 19;15:33

86. [Pectasides D](https://www-ncbi-nlm-nih-gov.gate2.inist.fr/pubmed/?term=Pectasides%20D%5BAuthor%5D&cauthor=true&cauthor_uid=25956750), [Karavasilis V](https://www-ncbi-nlm-nih-gov.gate2.inist.fr/pubmed/?term=Karavasilis%20V%5BAuthor%5D&cauthor=true&cauthor_uid=25956750), [Papaxoinis G](https://www-ncbi-nlm-nih-gov.gate2.inist.fr/pubmed/?term=Papaxoinis%20G%5BAuthor%5D&cauthor=true&cauthor_uid=25956750), et al. Randomized phase III clinical trial comparing the combination of capecitabine and oxaliplatin (CAPOX) with the combination of 5-fluorouracil, leucovorin and oxaliplatin (modified FOLFOX6) as adjuvant therapy in patients with operated high-risk stage II or stage III colorectal cancer. [BMC Cancer.](https://www-ncbi-nlm-nih-gov.gate2.inist.fr/pubmed/?term=25956750) 2015 May 10;15:384

87. [Pedersen P](https://www-ncbi-nlm-nih-gov.gate2.inist.fr/pubmed/?term=Pedersen%20P%5BAuthor%5D&cauthor=true&cauthor_uid=26253219), [Søgaard HJ](https://www-ncbi-nlm-nih-gov.gate2.inist.fr/pubmed/?term=S%C3%B8gaard%20HJ%5BAuthor%5D&cauthor=true&cauthor_uid=26253219), [Labriola M](https://www-ncbi-nlm-nih-gov.gate2.inist.fr/pubmed/?term=Labriola%20M%5BAuthor%5D&cauthor=true&cauthor_uid=26253219), et al. Effectiveness of psychoeducation in reducing sickness absence and improving mental health in individuals at risk of having a mental disorder: a randomised controlled trial. [BMC Public Health.](https://www-ncbi-nlm-nih-gov.gate2.inist.fr/pubmed/?term=26253219) 2015 Aug 8;15:763

88. [Peng W](https://www.ncbi.nlm.nih.gov/pubmed/?term=Peng%20W%5BAuthor%5D&cauthor=true&cauthor_uid=26464000), [Zhang T](https://www.ncbi.nlm.nih.gov/pubmed/?term=Zhang%20T%5BAuthor%5D&cauthor=true&cauthor_uid=26464000). Dexmedetomidine decreases the emergence agitation in infant patients undergoing cleft palate repair surgery after general anesthesia. [BMC Anesthesiol.](https://www.ncbi.nlm.nih.gov/pubmed/?term=26464000) 2015 Oct 13;15:145.

89. [Pontes MV](https://www-ncbi-nlm-nih-gov.gate2.inist.fr/pubmed/?term=Pontes%20MV%5BAuthor%5D&cauthor=true&cauthor_uid=26920136), [Ribeiro TC](https://www-ncbi-nlm-nih-gov.gate2.inist.fr/pubmed/?term=Ribeiro%20TC%5BAuthor%5D&cauthor=true&cauthor_uid=26920136), [Ribeiro H](https://www-ncbi-nlm-nih-gov.gate2.inist.fr/pubmed/?term=Ribeiro%20H%5BAuthor%5D&cauthor=true&cauthor_uid=26920136), et al. Cow's milk-based beverage consumption in 1- to 4-year-olds and allergic manifestations: an RCT. [Nutr J.](https://www-ncbi-nlm-nih-gov.gate2.inist.fr/pubmed/?term=26920136) 2016 Feb 27;15:19

90. [Pool D](https://www-ncbi-nlm-nih-gov.gate2.inist.fr/pubmed/?term=Pool%20D%5BAuthor%5D&cauthor=true&cauthor_uid=26459358) [Valentine J](https://www-ncbi-nlm-nih-gov.gate2.inist.fr/pubmed/?term=Valentine%20J%5BAuthor%5D&cauthor=true&cauthor_uid=26459358), [Bear N](https://www-ncbi-nlm-nih-gov.gate2.inist.fr/pubmed/?term=Bear%20N%5BAuthor%5D&cauthor=true&cauthor_uid=26459358), [Donnelly CJ](https://www-ncbi-nlm-nih-gov.gate2.inist.fr/pubmed/?term=Donnelly%20CJ%5BAuthor%5D&cauthor=true&cauthor_uid=26459358), et al. The orthotic and therapeutic effects following daily community applied functional electrical stimulation in children with unilateral spastic cerebral palsy: a randomised controlled trial. [BMC Pediatr.](https://www-ncbi-nlm-nih-gov.gate2.inist.fr/pubmed/?term=The+orthotic+and+therapeutic+effects+following+daily+community+applied+functional+electrical+stimulation+in+children+with+unilateral+spastic+cerebral+palsy%3A+a+randomised+controlled+trial) 2015 Oct 12;15:154

91. [Rasmussen KC](https://www-ncbi-nlm-nih-gov.gate2.inist.fr/pubmed/?term=Rasmussen%20KC%5BAuthor%5D&cauthor=true&cauthor_uid=26646213), [Hoejskov M](https://www-ncbi-nlm-nih-gov.gate2.inist.fr/pubmed/?term=Hoejskov%20M%5BAuthor%5D&cauthor=true&cauthor_uid=26646213), [Johansson PI](https://www-ncbi-nlm-nih-gov.gate2.inist.fr/pubmed/?term=Johansson%20PI%5BAuthor%5D&cauthor=true&cauthor_uid=26646213), et al. Coagulation competence for predicting perioperative hemorrhage in patients treated with lactated Ringer's vs. Dextran--a randomized controlled trial. [BMC Anesthesiol.](https://www-ncbi-nlm-nih-gov.gate2.inist.fr/pubmed/?term=26646213) 2015 Dec 8;15:178

92. [Rixen D](https://www-ncbi-nlm-nih-gov.gate2.inist.fr/pubmed/?term=Rixen%20D%5BAuthor%5D&cauthor=true&cauthor_uid=26809247), [Steinhausen E](https://www-ncbi-nlm-nih-gov.gate2.inist.fr/pubmed/?term=Steinhausen%20E%5BAuthor%5D&cauthor=true&cauthor_uid=26809247), [Sauerland S](https://www-ncbi-nlm-nih-gov.gate2.inist.fr/pubmed/?term=Sauerland%20S%5BAuthor%5D&cauthor=true&cauthor_uid=26809247), et al. Randomized, controlled, two-arm, interventional, multicenter study on risk-adapted damage control orthopedic surgery of femur shaft fractures in multiple-trauma patients. [Trials.](https://www-ncbi-nlm-nih-gov.gate2.inist.fr/pubmed/?term=26809247) 2016 Jan 25;17:47

93. [Rongsen-Chandola T](https://www-ncbi-nlm-nih-gov.gate2.inist.fr/pubmed/?term=Rongsen-Chandola%20T%5BAuthor%5D&cauthor=true&cauthor_uid=24976452), [Winje BA](https://www-ncbi-nlm-nih-gov.gate2.inist.fr/pubmed/?term=Winje%20BA%5BAuthor%5D&cauthor=true&cauthor_uid=24976452), [Goyal N](https://www-ncbi-nlm-nih-gov.gate2.inist.fr/pubmed/?term=Goyal%20N%5BAuthor%5D&cauthor=true&cauthor_uid=24976452), et al. Compliance of mothers following recommendations to breastfeed or withholdbreast milk during rotavirus vaccination in North India: a randomized clinical trial. [Trials.](https://www-ncbi-nlm-nih-gov.gate2.inist.fr/pubmed/24976452) 2014 Jun 28;15:256

94. [Salaffi F](https://www-ncbi-nlm-nih-gov.gate2.inist.fr/pubmed/?term=Salaffi%20F%5BAuthor%5D&cauthor=true&cauthor_uid=27038788), [Carotti M](https://www-ncbi-nlm-nih-gov.gate2.inist.fr/pubmed/?term=Carotti%20M%5BAuthor%5D&cauthor=true&cauthor_uid=27038788), [Ciapetti A](https://www-ncbi-nlm-nih-gov.gate2.inist.fr/pubmed/?term=Ciapetti%20A%5BAuthor%5D&cauthor=true&cauthor_uid=27038788), et al. Effectiveness of a telemonitoring intensive strategy in early rheumatoid arthritis: comparison with the conventional management approach. [BMC Musculoskelet Disord.](https://www-ncbi-nlm-nih-gov.gate2.inist.fr/pubmed/27038788) 2016 Apr 2;17:146

95. [Schachtel B](https://www-ncbi-nlm-nih-gov.gate2.inist.fr/pubmed/?term=Schachtel%20B%5BAuthor%5D&cauthor=true&cauthor_uid=24988909), [Aspley S](https://www-ncbi-nlm-nih-gov.gate2.inist.fr/pubmed/?term=Aspley%20S%5BAuthor%5D&cauthor=true&cauthor_uid=24988909), [Shephard A](https://www-ncbi-nlm-nih-gov.gate2.inist.fr/pubmed/?term=Shephard%20A%5BAuthor%5D&cauthor=true&cauthor_uid=24988909), et al. Utility of the sore throat pain model in a multiple-dose assessment of the acute analgesic flurbiprofen: a randomized controlled study. [Trials.](https://www-ncbi-nlm-nih-gov.gate2.inist.fr/pubmed/?term=Utility+of+the+sore+throat+pain+model+in+a+multiple-dose+assessment+of+the+acute+analgesic+flurbiprofen%3A+a+randomized+controlled+study) 2014 Jul 3;15:263

96. [Schaffer JT](https://www-ncbi-nlm-nih-gov.gate2.inist.fr/pubmed/?term=Schaffer%20JT%5BAuthor%5D&cauthor=true&cauthor_uid=25577713), [Hunter BR](https://www-ncbi-nlm-nih-gov.gate2.inist.fr/pubmed/?term=Hunter%20BR%5BAuthor%5D&cauthor=true&cauthor_uid=25577713), [Ball KM](https://www-ncbi-nlm-nih-gov.gate2.inist.fr/pubmed/?term=Ball%20KM%5BAuthor%5D&cauthor=true&cauthor_uid=25577713), et al. Noninvasive sphenopalatine ganglion block for acute headache in the emergency department: a randomized placebo-controlled trial. [Ann Emerg Med.](https://www-ncbi-nlm-nih-gov.gate2.inist.fr/pubmed/?term=25577713) 2015 May;65(5):503-10

97. [Sedkaoui K](https://www-ncbi-nlm-nih-gov.gate2.inist.fr/pubmed/?term=Sedkaoui%20K%5BAuthor%5D&cauthor=true&cauthor_uid=26370444), [Leseux L](https://www-ncbi-nlm-nih-gov.gate2.inist.fr/pubmed/?term=Leseux%20L%5BAuthor%5D&cauthor=true&cauthor_uid=26370444), [Pontier S](https://www-ncbi-nlm-nih-gov.gate2.inist.fr/pubmed/?term=Pontier%20S%5BAuthor%5D&cauthor=true&cauthor_uid=26370444), et al. Efficiency of a phone coaching program on adherence to continuous positive airway pressure in sleep apnea hypopnea syndrome: a randomized trial. [BMC Pulm Med.](https://www-ncbi-nlm-nih-gov.gate2.inist.fr/pubmed/?term=26370444) 2015 Sep 14;15:102

98. [Singh D](https://www-ncbi-nlm-nih-gov.gate2.inist.fr/pubmed/?term=Singh%20D%5BAuthor%5D&cauthor=true&cauthor_uid=26286141), [Worsley S](https://www-ncbi-nlm-nih-gov.gate2.inist.fr/pubmed/?term=Worsley%20S%5BAuthor%5D&cauthor=true&cauthor_uid=26286141), [Zhu CQ](https://www-ncbi-nlm-nih-gov.gate2.inist.fr/pubmed/?term=Zhu%20CQ%5BAuthor%5D&cauthor=true&cauthor_uid=26286141), et al. Umeclidinium/vilanterol versus fluticasone propionate/salmeterol in COPD: a randomised trial. [BMC Pulm Med.](https://www-ncbi-nlm-nih-gov.gate2.inist.fr/pubmed/?term=26286141%22) 2015 Aug 19;15:91

99. [Smith JE](https://www-ncbi-nlm-nih-gov.gate2.inist.fr/pubmed/?term=Smith%20JE%5BAuthor%5D&cauthor=true&cauthor_uid=26094763), [Rockett M](https://www-ncbi-nlm-nih-gov.gate2.inist.fr/pubmed/?term=Rockett%20M%5BAuthor%5D&cauthor=true&cauthor_uid=26094763), [S SC](https://www-ncbi-nlm-nih-gov.gate2.inist.fr/pubmed/?term=S%20SC%5BAuthor%5D&cauthor=true&cauthor_uid=26094763), et al. PAin SoluTions In the Emergency Setting (PASTIES)--patient controlled analgesia versus routine care in emergency department patients with pain from traumatic injuries: randomised trial. [BMJ.](https://www-ncbi-nlm-nih-gov.gate2.inist.fr/pubmed/?term=26094763%22) 2015 Jun 21;350:h2988

100. [Sun BC](https://www-ncbi-nlm-nih-gov.gate2.inist.fr/pubmed/?term=Sun%20BC%5BAuthor%5D&cauthor=true&cauthor_uid=24239341), [McCreath H](https://www-ncbi-nlm-nih-gov.gate2.inist.fr/pubmed/?term=McCreath%20H%5BAuthor%5D&cauthor=true&cauthor_uid=24239341), [Liang LJ](https://www-ncbi-nlm-nih-gov.gate2.inist.fr/pubmed/?term=Liang%20LJ%5BAuthor%5D&cauthor=true&cauthor_uid=24239341), et al. Randomized clinical trial of an emergency department observation syncope protocol versus routine inpatient admission. [Ann Emerg Med.](https://www-ncbi-nlm-nih-gov.gate2.inist.fr/pubmed/?term=24239341) 2014 Aug;64(2):167-75

101. [Takahashi H](https://www-ncbi-nlm-nih-gov.gate2.inist.fr/pubmed/?term=Takahashi%20H%5BAuthor%5D&cauthor=true&cauthor_uid=25609176), [Arimura Y](https://www-ncbi-nlm-nih-gov.gate2.inist.fr/pubmed/?term=Arimura%20Y%5BAuthor%5D&cauthor=true&cauthor_uid=25609176), [Okahara S](https://www-ncbi-nlm-nih-gov.gate2.inist.fr/pubmed/?term=Okahara%20S%5BAuthor%5D&cauthor=true&cauthor_uid=25609176), et al. A randomized controlled trial of endoscopic steroid injection for prophylaxis of esophageal stenoses after extensive endoscopic submucosal dissection. [BMC Gastroenterol.](https://www-ncbi-nlm-nih-gov.gate2.inist.fr/pubmed/?term=25609176) 2015 Jan 22;15:1

102. [Taraldsen K](https://www-ncbi-nlm-nih-gov.gate2.inist.fr/pubmed/?term=Taraldsen%20K%5BAuthor%5D&cauthor=true&cauthor_uid=26637222), [Thingstad P](https://www-ncbi-nlm-nih-gov.gate2.inist.fr/pubmed/?term=Thingstad%20P%5BAuthor%5D&cauthor=true&cauthor_uid=26637222), [Sletvold O](https://www-ncbi-nlm-nih-gov.gate2.inist.fr/pubmed/?term=Sletvold%20O%5BAuthor%5D&cauthor=true&cauthor_uid=26637222), et al. The long-term effect of being treated in a geriatric ward compared to an orthopaedic ward on six measures of free-living physical behavior 4 and 12 months after a hip fracture - a randomised controlled trial. [BMC Geriatr.](https://www-ncbi-nlm-nih-gov.gate2.inist.fr/pubmed/?term=26637222) 2015 Dec 4;15:160

103. [Thybo KH](https://www-ncbi-nlm-nih-gov.gate2.inist.fr/pubmed/?term=Thybo%20KH%5BAuthor%5D&cauthor=true&cauthor_uid=27006014), [Schmidt H](https://www-ncbi-nlm-nih-gov.gate2.inist.fr/pubmed/?term=Schmidt%20H%5BAuthor%5D&cauthor=true&cauthor_uid=27006014), [Hägi-Pedersen D](https://www-ncbi-nlm-nih-gov.gate2.inist.fr/pubmed/?term=H%C3%A4gi-Pedersen%20D%5BAuthor%5D&cauthor=true&cauthor_uid=27006014). Effect of lateral femoral cutaneous nerve-block on pain after total hip arthroplasty: a randomised, blinded, placebo-controlled trial. [BMC Anesthesiol.](https://www-ncbi-nlm-nih-gov.gate2.inist.fr/pubmed/?term=27006014) 2016 Mar 23;16:21.

104. [Traub SJ](https://www-ncbi-nlm-nih-gov.gate2.inist.fr/pubmed/?term=Traub%20SJ%5BAuthor%5D&cauthor=true&cauthor_uid=23769807), [Mitchell AM](https://www-ncbi-nlm-nih-gov.gate2.inist.fr/pubmed/?term=Mitchell%20AM%5BAuthor%5D&cauthor=true&cauthor_uid=23769807), [Jones AE](https://www-ncbi-nlm-nih-gov.gate2.inist.fr/pubmed/?term=Jones%20AE%5BAuthor%5D&cauthor=true&cauthor_uid=23769807), et al. N-acetylcysteine plus intravenous fluids versus intravenous fluids alone to prevent contrast-induced nephropathy in emergency computed tomography. [Ann Emerg Med.](https://www-ncbi-nlm-nih-gov.gate2.inist.fr/pubmed/?term=23769807) 2013 Nov;62(5):511-520.e25

105. [Travier N](https://www-ncbi-nlm-nih-gov.gate2.inist.fr/pubmed/?term=Travier%20N%5BAuthor%5D&cauthor=true&cauthor_uid=26050790), [Velthuis MJ](https://www-ncbi-nlm-nih-gov.gate2.inist.fr/pubmed/?term=Velthuis%20MJ%5BAuthor%5D&cauthor=true&cauthor_uid=26050790), [Steins Bisschop CN](https://www-ncbi-nlm-nih-gov.gate2.inist.fr/pubmed/?term=Steins%20Bisschop%20CN%5BAuthor%5D&cauthor=true&cauthor_uid=26050790), et al. Effects of an 18-week exercise programme started early during breast cancer treatment: a randomised controlled trial. [BMC Med.](https://www-ncbi-nlm-nih-gov.gate2.inist.fr/pubmed/?term=26050790) 2015 Jun 8;13:121

106. [Tumukunde J](https://www-ncbi-nlm-nih-gov.gate2.inist.fr/pubmed/?term=Tumukunde%20J%5BAuthor%5D&cauthor=true&cauthor_uid=25924776), [Lomangisi DD](https://www-ncbi-nlm-nih-gov.gate2.inist.fr/pubmed/?term=Lomangisi%20DD%5BAuthor%5D&cauthor=true&cauthor_uid=25924776), [Davidson O](https://www-ncbi-nlm-nih-gov.gate2.inist.fr/pubmed/?term=Davidson%20O%5BAuthor%5D&cauthor=true&cauthor_uid=25924776), et al. Effects of propofol versus thiopental on Apgar scores in newborns and peri-operative outcomes of women undergoing emergency cesarean section: a randomized clinical trial. [BMC Anesthesiol.](https://www-ncbi-nlm-nih-gov.gate2.inist.fr/pubmed/?term=25924776) 2015 Apr 29;15:63

107. [Tuntland H](https://www-ncbi-nlm-nih-gov.gate2.inist.fr/pubmed/?term=Tuntland%20H%5BAuthor%5D&cauthor=true&cauthor_uid=26537789), [Aaslund MK](https://www-ncbi-nlm-nih-gov.gate2.inist.fr/pubmed/?term=Aaslund%20MK%5BAuthor%5D&cauthor=true&cauthor_uid=26537789), [Espehaug B](https://www-ncbi-nlm-nih-gov.gate2.inist.fr/pubmed/?term=Espehaug%20B%5BAuthor%5D&cauthor=true&cauthor_uid=26537789), et al. Reablement in community-dwelling older adults: a randomised controlled trial. [BMC Geriatr.](https://www-ncbi-nlm-nih-gov.gate2.inist.fr/pubmed/?term=26537789) 2015 Nov 4;15:145

108. [Unger HW](https://www-ncbi-nlm-nih-gov.gate2.inist.fr/pubmed/?term=Unger%20HW%5BAuthor%5D&cauthor=true&cauthor_uid=25591391), [Ome-Kaius M](https://www-ncbi-nlm-nih-gov.gate2.inist.fr/pubmed/?term=Ome-Kaius%20M%5BAuthor%5D&cauthor=true&cauthor_uid=25591391), [Wangnapi RA](https://www-ncbi-nlm-nih-gov.gate2.inist.fr/pubmed/?term=Wangnapi%20RA%5BAuthor%5D&cauthor=true&cauthor_uid=25591391), et al. Sulphadoxine-pyrimethamine plus azithromycin for the prevention of low birthweight in Papua New Guinea: a randomised controlled trial. [BMC Med.](https://www-ncbi-nlm-nih-gov.gate2.inist.fr/pubmed/?term=25591391) 2015 Jan 16;13:9

109. [van der Aa HP](https://www-ncbi-nlm-nih-gov.gate2.inist.fr/pubmed/?term=van%20der%20Aa%20HP%5BAuthor%5D&cauthor=true&cauthor_uid=26597263), [van Rens GH](https://www-ncbi-nlm-nih-gov.gate2.inist.fr/pubmed/?term=van%20Rens%20GH%5BAuthor%5D&cauthor=true&cauthor_uid=26597263), [Comijs HC](https://www-ncbi-nlm-nih-gov.gate2.inist.fr/pubmed/?term=Comijs%20HC%5BAuthor%5D&cauthor=true&cauthor_uid=26597263), et al. Stepped care for depression and anxiety in visually impaired older adults: multicentre randomised controlled trial. [BMJ.](https://www-ncbi-nlm-nih-gov.gate2.inist.fr/pubmed/?term=26597263) 2015 Nov 23;351:h6127

110. [Viasus D](https://www-ncbi-nlm-nih-gov.gate2.inist.fr/pubmed/?term=Viasus%20D%5BAuthor%5D&cauthor=true&cauthor_uid=25564143), [Garcia-Vidal C](https://www-ncbi-nlm-nih-gov.gate2.inist.fr/pubmed/?term=Garcia-Vidal%20C%5BAuthor%5D&cauthor=true&cauthor_uid=25564143), [Simonetti AF](https://www-ncbi-nlm-nih-gov.gate2.inist.fr/pubmed/?term=Simonetti%20AF%5BAuthor%5D&cauthor=true&cauthor_uid=25564143), et al. The effect of simvastatin on inflammatory cytokines in community-acquired pneumonia: a randomised, double-blind, placebo-controlled trial. [BMJ Open.](https://www-ncbi-nlm-nih-gov.gate2.inist.fr/pubmed/?term=25564143) 2015 Jan 6;5(1):e006251

111. [Wang L](https://www-ncbi-nlm-nih-gov.gate2.inist.fr/pubmed/?term=Wang%20L%5BAuthor%5D&cauthor=true&cauthor_uid=25879863), [Zhong Z](https://www-ncbi-nlm-nih-gov.gate2.inist.fr/pubmed/?term=Zhong%20Z%5BAuthor%5D&cauthor=true&cauthor_uid=25879863), [Hu J](https://www-ncbi-nlm-nih-gov.gate2.inist.fr/pubmed/?term=Hu%20J%5BAuthor%5D&cauthor=true&cauthor_uid=25879863), et al. Sertraline plus deanxit to treat patients with depression and anxiety in chronic somatic diseases: a randomized controlled trial. [BMC Psychiatry.](https://www-ncbi-nlm-nih-gov.gate2.inist.fr/pubmed/?term=25879863) 2015 Apr 14;15:84

112. [Weeks AD](https://www-ncbi-nlm-nih-gov.gate2.inist.fr/pubmed/?term=Weeks%20AD%5BAuthor%5D&cauthor=true&cauthor_uid=26370443), [Ditai J](https://www-ncbi-nlm-nih-gov.gate2.inist.fr/pubmed/?term=Ditai%20J%5BAuthor%5D&cauthor=true&cauthor_uid=26370443), [Ononge S](https://www-ncbi-nlm-nih-gov.gate2.inist.fr/pubmed/?term=Ononge%20S%5BAuthor%5D&cauthor=true&cauthor_uid=26370443), et al. The MamaMiso study of self-administered misoprostol to prevent bleeding after childbirth in rural Uganda: a community-based, placebo-controlled randomised trial. [BMC Pregnancy Childbirth.](https://www-ncbi-nlm-nih-gov.gate2.inist.fr/pubmed/?term=26370443) 2015 Sep 14;15:219

113. [Wilson AM](https://www-ncbi-nlm-nih-gov.gate2.inist.fr/pubmed/?term=Wilson%20AM%5BAuthor%5D&cauthor=true&cauthor_uid=25762226), [Browne P](https://www-ncbi-nlm-nih-gov.gate2.inist.fr/pubmed/?term=Browne%20P%5BAuthor%5D&cauthor=true&cauthor_uid=25762226), [Olive S](https://www-ncbi-nlm-nih-gov.gate2.inist.fr/pubmed/?term=Olive%20S%5BAuthor%5D&cauthor=true&cauthor_uid=25762226), et al. The effects of maintenance schedules following pulmonary rehabilitation in patients with chronic obstructive pulmonary disease: a randomised controlled trial. [BMJ Open.](https://www-ncbi-nlm-nih-gov.gate2.inist.fr/pubmed/?term=25762226) 2015 Mar 11;5(3):e005921

114. [Yang YG](https://www-ncbi-nlm-nih-gov.gate2.inist.fr/pubmed/?term=Yang%20YG%5BAuthor%5D&cauthor=true&cauthor_uid=26547293), [Hu LH](https://www-ncbi-nlm-nih-gov.gate2.inist.fr/pubmed/?term=Hu%20LH%5BAuthor%5D&cauthor=true&cauthor_uid=26547293), [Chen H](https://www-ncbi-nlm-nih-gov.gate2.inist.fr/pubmed/?term=Chen%20H%5BAuthor%5D&cauthor=true&cauthor_uid=26547293), et al. Target-controlled infusion of remifentanil with or without flurbiprofen axetil in sedation for extracorporeal shock wave lithotripsy of pancreatic stones: a prospective, open-label, randomized controlled trial. [BMC Anesthesiol.](https://www-ncbi-nlm-nih-gov.gate2.inist.fr/pubmed/?term=26547293) 2015 Nov 7;15:161

115. [Yi J](https://www.ncbi.nlm.nih.gov/pubmed/?term=Yi%20J%5BAuthor%5D&cauthor=true&cauthor_uid=25927657), [Gong Y](https://www.ncbi.nlm.nih.gov/pubmed/?term=Gong%20Y%5BAuthor%5D&cauthor=true&cauthor_uid=25927657), [Quan X](https://www.ncbi.nlm.nih.gov/pubmed/?term=Quan%20X%5BAuthor%5D&cauthor=true&cauthor_uid=25927657), et al. Comparison of the Airtraq laryngoscope and the GlideScope for double-lumen tube intubation in patients with predicted normal airways: a prospective randomized trial. [BMC Anesthesiol.](https://www.ncbi.nlm.nih.gov/pubmed/?term=25927657%5Buid%5D) 2015 Apr 28;15:58.

116. [Yokoyama H](https://www-ncbi-nlm-nih-gov.gate2.inist.fr/pubmed/?term=Yokoyama%20H%5BAuthor%5D&cauthor=true&cauthor_uid=26018225), [Okazaki K](https://www-ncbi-nlm-nih-gov.gate2.inist.fr/pubmed/?term=Okazaki%20K%5BAuthor%5D&cauthor=true&cauthor_uid=26018225), [Imai D](https://www-ncbi-nlm-nih-gov.gate2.inist.fr/pubmed/?term=Imai%20D%5BAuthor%5D&cauthor=true&cauthor_uid=26018225), et al. The effect of cognitive-motor dual-task training on cognitive function and plasma amyloid β peptide 42/40 ratio in healthy elderly persons: a randomized controlled trial.

[BMC Geriatr.](https://www-ncbi-nlm-nih-gov.gate2.inist.fr/pubmed/?term=26018225) 2015 May 28;15:60

117. [Zaballos M](https://www-ncbi-nlm-nih-gov.gate2.inist.fr/pubmed/?term=Zaballos%20M%5BAuthor%5D&cauthor=true&cauthor_uid=26438179), [Bastida E](https://www-ncbi-nlm-nih-gov.gate2.inist.fr/pubmed/?term=Bastida%20E%5BAuthor%5D&cauthor=true&cauthor_uid=26438179), [Agustí S](https://www-ncbi-nlm-nih-gov.gate2.inist.fr/pubmed/?term=Agust%C3%AD%20S%5BAuthor%5D&cauthor=true&cauthor_uid=26438179), et al. Effect-site concentration of propofol required for LMA-Supreme™ insertion with and without remifentanil: a randomized controlled trial. [BMC Anesthesiol.](https://www-ncbi-nlm-nih-gov.gate2.inist.fr/pubmed/?term=26438179%22) 2015 Oct 6;15:131

118. [Zhang L](https://www-ncbi-nlm-nih-gov.gate2.inist.fr/pubmed/?term=Zhang%20L%5BAuthor%5D&cauthor=true&cauthor_uid=26071690), [Liu Z](https://www-ncbi-nlm-nih-gov.gate2.inist.fr/pubmed/?term=Liu%20Z%5BAuthor%5D&cauthor=true&cauthor_uid=26071690), [Wang J](https://www-ncbi-nlm-nih-gov.gate2.inist.fr/pubmed/?term=Wang%20J%5BAuthor%5D&cauthor=true&cauthor_uid=26071690), et al. Unipedicular versus bipedicular percutaneous vertebroplasty for osteoporotic vertebral compression fractures: a prospective randomized study. [BMC Musculoskelet Disord.](https://www-ncbi-nlm-nih-gov.gate2.inist.fr/pubmed/?term=26071690) 2015 Jun 14;16:145

119. [Zhou SJ](https://www-ncbi-nlm-nih-gov.gate2.inist.fr/pubmed/?term=Zhou%20SJ%5BAuthor%5D&cauthor=true&cauthor_uid=26654905), [Skeaff SA](https://www-ncbi-nlm-nih-gov.gate2.inist.fr/pubmed/?term=Skeaff%20SA%5BAuthor%5D&cauthor=true&cauthor_uid=26654905), [Ryan P](https://www-ncbi-nlm-nih-gov.gate2.inist.fr/pubmed/?term=Ryan%20P%5BAuthor%5D&cauthor=true&cauthor_uid=26654905), et al. The effect of iodine supplementation in pregnancy on early childhood neurodevelopment and clinical outcomes: results of an aborted randomised placebo-controlled trial. [Trials.](https://www-ncbi-nlm-nih-gov.gate2.inist.fr/pubmed/?term=26654905) 2015 Dec 10;16:563
