# Supplementary material for: Accuracy in detecting inadequate research reporting by early career peer reviewers using an online CONSORT-based peer-review tool (COBPeer) versus the usual peer-review process: a cross-sectional diagnostic study
Source: BMC Med. 2019 Nov 19;17:205. doi: 10.1186/s12916-019-1436-0 (PMC6864983; doi:10.1186/s12916-019-1436-0)
Supplement: Supplementary file 5 — Additional file 5. The summary of the process. Full details of the design of the COBPeer study. [file 12916_2019_1436_MOESM5_ESM.docx]

Additional files 5. The summary of the process

**119 selected manuscripts**

**(initial submitted report)**

**Group 1 - Usual peer-review process**

- We considered and merged all the peer-review reports performed by peer-reviewers during the first round of the peer-review process
- Peer-reviewers were not aware that their reports will be used in this study
- They were aware that it was an open-peer-review process and that their report would be made available on the journal website.
- They were not specifically prompted to assess the reporting of the CONSORT items although journals selected all endorsed CONSORT.
- They were not specifically trained to evaluate CONSORT items.
- Their assessment was performed before the study was launched and they had no access to COBPeer.
- They were expected to perform several tasks such as evaluating the relevance of the question, the methods, the interpretation, reporting etc.
- The usual peer-reviewers’ assessments were extracted by 2 senior clinical epidemiologists. They were blinded to the reference standard and ECR assessments and they did not use COBPeer. They used an online standardized data extraction form available at (https://forms.gle/ybykYbFanFu1nB8z9). They were not involved in other data extractions. When one peer-reviewer mentioned inadequate reporting for a specific CONSORT item, the item was considered incompletely reported.

**Group 2 - ECR peer-reviewers assessment**

- Each manuscript was evaluated by a single ECR peer-reviewer.
- ECRs were aware that they were participating in a study.
- They were specifically trained in using COBPeer and identifying incomplete reporting.
- They had a unique task to perform: assess the completeness of reporting with COBPeer.
- Their assessment was performed online and directly downloaded for analysis.

**Reference standard - expert systematic reviewers**

- Each manuscript was evaluated independently by 4 pairs of expert systematic reviewers with consensus achieved in case of disagreement.
- The expert systematic reviewers involved did not participate in the assessment of the peer-review reports and were blinded to the results of the assessment by the usual peer-review process and ECR peer-reviewers.
- Expert systematic reviewers used an online data extraction form (https://forms.gle/5RzWhGG6rEwkJxoo7).

The 119 first analysis by ECR was included in the analysis

Assessment by 2 independent researchers (**standard** **peer-review**):

1. The completeness of reporting

2. Switched outcomes

Assessment by 1 pair of systematic reviewers among 4 pairs of systematic reviewers:

1. The completeness of reporting

2. Switched outcomes

131 ECR assessed a report **with the COBPeer tool**:

1. The completeness of reporting

2. Switched outcomes
