# Supplementary material for: Accuracy in detecting inadequate research reporting by early career peer reviewers using an online CONSORT-based peer-review tool (COBPeer) versus the usual peer-review process: a cross-sectional diagnostic study
Source: BMC Med. 2019 Nov 19;17:205. doi: 10.1186/s12916-019-1436-0 (PMC6864983; doi:10.1186/s12916-019-1436-0)
Supplement: Supplementary file 6 — Additional file 6. The COBPeer tool. Details of each domains evaluated by early career researchers with COBPeer tool. [file 12916_2019_1436_MOESM6_ESM.docx]

Additional file 6. The COBPeer tool

| **CONSORT Item** | **CONSORT item** | | | |
| --- | --- | --- | --- | --- |
| **Outcomes** | **Item 6a. Completely defined pre-specified primary outcome measures, including how and when they were assessed**^[[1]](#footnote-1)^ | | | |
|  |  | | **Yes** | **No** |
|  | **Was the primary outcome(s) clearly identified (e.g., the primary/main outcome was pain)?** | |  |  |
|  | **If no go to next section**  **If yes answer the following questions,**  **Please check if the author clearly report for the primary outcome(s):** | |  |  |
|  | - The variable of interest (e.g., pain, all-cause mortality) | |  |  |
|  | - How the outcome was assessed (e.g., VAS, Beck Depression Inventory score, pain scale) | |  |  |
|  | - The analysis metric (e.g., change from baseline, final value, time to event) | |  |  |
|  | - The summary measure for each study group (e.g., mean, proportion with score > 2) | |  |  |
|  | - Time point of interest for analysis (e.g., 3 months)****NA if survival analysis*** | |  |  |
|  | - Who assessed the outcome (e.g., the patient, doctor, nurse, caregiver, other) | |  |  |
| **Randomization**  **Sequence generation** | **Item 8a. Method used to generate the random allocation sequence** | | | |
|  | **Did the author report:** | | **Yes** | **No** |
|  | - - The method of sequence generation (e.g., a random number table or computerized random number generator, or other) | |  |  |
| **Allocation concealment** | **Item 9. Mechanism used to implement the random allocation sequence (e.g., sequentially numbered containers), describing any steps taken to conceal the sequence until interventions were assigned** | | | |
|  | **Did the author report:** | | **Yes** | **No** |
|  | - - How the care provider enrolling patients was blinded to the next assignment in the sequence. Possible methods can rely on     - For centralised or “third-party” assignment (i.e, use of a central telephone randomization system, automated assignment system)     - Having a third party prepare the randomization list and hide the allocation assignment in advance via numbered identical bottles or sequentially numbered, sealed, opaque envelopes     - If the mechanism of the random allocation sequence is completely described but the sequence is not adequately concealed, please tick yes | |  |  |
|  | Was the study blinded yes/no  - If yes go to 11a - If no go to 13a | | | |
| **Blinding** | Item 11a. If done, who was blinded after assignment to interventions (e.g., participants, care providers, those assessing outcomes) and how | | | |
|  | Item 11b. If relevant, description of the similarity of interventions | | | |
|  | **Did the author report:** | | **Yes** | **No** |
|  | - - Who (i.e., participants, healthcare providers, data collectors, outcome adjudicators, and data analysts) was blinded to treatment assignments? | |  |  |
|  | - - How was the blinding performed? (e.g., used of placebo, intervention by physician unaware of the study) | |  |  |
|  | - - The similarities of the characteristics of the interventions (e.g., appearance, taste, smell, method of administration)****NA*** | |  |  |
| **Participant flow** | **Item 13a. For each group, the numbers of participants who were randomly assigned, received intended treatment, and were analyzed for the primary outcome**  **Item 13b. For each group, losses and exclusions after randomization, together with reasons** | | | |
|  | **Did the authors report a flow chart** | | **Yes** | **No** |
|  |  | |  |  |
|  | **Did the author report in the flow chart or in the text:** | | **Yes** | **No** |
|  | - - Number of participants randomized in each group | |  |  |
|  | - - Number of participants who received intended treatment in each group | |  |  |
|  | - - Number of participants who did not receive the allocated treatment with reasons in each group | |  |  |
|  | - - Number of participants lost to follow-up with reasons in each group | |  |  |
|  | - - Number of participants who discontinued intervention with reasons in each group | |  |  |
|  | - - Number of participants analyzed for the primary outcome in each group | |  |  |
|  | - - Number excluded from analysis with reasons in each group | |  |  |
| **Outcomes and estimation** | **Item 17a. For each primary outcome, results for each group, and the estimated effect size and its precision (such as 95% confidence interval)** | | | |
|  | **Did the author report for primary outcome: (answer yes if true for all primary outcomes)** | | **Yes** | **No** |
|  | - - Result in each group (mean (SD) or number of events/N) | |  |  |
|  | - - Difference in estimated effect between groups (e.g., odds ratio (OR), risk ratio (RR), risk difference (RD), hazard ratio (HR), difference in median survival time, mean difference (MD)) | |  |  |
|  | - - Precision for difference between groups (e.g., 95% CI) | |  |  |
| **Harms** | **Item 19. All-important harms or unintended effects in each group** | | | |
|  | **Did the author report:** | | **Yes** | **No** |
|  | - - How harms-related information was collected (e.g., mode of data collection, timing, attribution methods) | |  |  |
|  | - - For each group, participant withdrawals due to harm | |  |  |
|  | - - Results in each group for each harms type with denominator (mean [SD] or number of events/N) | |  |  |
| **Registration** | **Item 23. Registration number and name of registry** | | | |
|  | **Did the author report:** | | **Yes** | **No** |
|  | - - The registration number | |  |  |
| **Consistency between data registered and reported**  **Did authors report the same primary outcome in the register and manuscript (same variable, same metric, same time point) or was the primary outcome added, deleted, changed** | | | | |
| **If the study was not registered, please tick the box □** | | | | |
| **If the study was registered, report** | | | | |
| - **The link to the online registry:** | | | | |
| - **Date of the registration:** | | | | |
| - **Date of the start of the study:** | | | | |
| **Was the primary outcome(s) reported in the register or manuscript not sufficiently described to identify a switch in outcomes?** | | | **Yes** | **No** |
| **Did you identify any outcome(s) reported by the authors as a primary outcome(s) but not registered as such?** | | | **Yes** | **No** |
| **Did you identify any outcome(s) registered as a primary outcome(s) but not reported as such in the manuscript?** | | | **Yes** | **No** |
| **Did you identify any change in terms of time frame, metric or other information between the primary outcome(s) registered and reported in the manuscript?** | | | **Yes** | **No** |
| **If yes, please list the discrepancies:** | | | | |
| - | | | | |
| - | | | | |
| **Did the authors justify the switched outcome(s) in the manuscript?** | | **Yes** | **No** | **NA** |
|  |  |  |  |  |

1. This CONSORT item was slightly modified to focus on the primary outcome. [↑](#footnote-ref-1)
