# Supplementary material for: Accuracy in detecting inadequate research reporting by early career peer reviewers using an online CONSORT-based peer-review tool (COBPeer) versus the usual peer-review process: a cross-sectional diagnostic study
Source: BMC Med. 2019 Nov 19;17:205. doi: 10.1186/s12916-019-1436-0 (PMC6864983; doi:10.1186/s12916-019-1436-0)
Supplement: Supplementary file 7 — Additional file 7. Information for participants. The invitation letter received by the early career researchers. [file 12916_2019_1436_MOESM7_ESM.docx]

Additional file 7. Information for participants

**INFORMATION FOR PARTICIPANTS**

**Title: Online Training Module to Peer Review.**

Dear colleague /XX/student,

We would like to invite you to participate in an academic study of an online training module and a specific tool dedicated to early career peer reviewers when assessing a randomized controlled trial.

This study is performed in collaboration with medical journal editors, the CONSORT group and the EQUATOR network.

When the study is completed, you will receive 1 credit of Continued Medical Education (CME) for your participation in this training programme. Further, we will send you the study results and acknowledge your participation, if you agree to have your name cited in the manuscript acknowledgments section. Finally, this programme is a unique opportunity for you to gain expertise in peer-reviewing and become a peer reviewer for journal editors.

This training programme is focused on the assessment of the completeness of reporting in manuscripts of randomized controlled trials according to the CONSORT statements, and the switch in primary outcomes, which are essential elements in the peer review process. During this programme, you will 1) assess the completeness of reporting of manuscripts extracts using the tool (with immediate feedback and explanation of incorrect responses), and 2) assess a full text article using the tool.

If you are successful (i.e. >80% appropriate answers), you will have to assess a final manuscript.

We estimate that this process with take about one hour.

All information collected during this study will be treated confidentially.

Thank you very much in advance for your help with this project

Sincerely,

Pr Isabelle Boutron (Paris Descartes University, INSERM, France),

Dr Anthony Chauvin (Paris Descartes University, INSERM, France)

Dr David Moher (Ottawa Hospital Research Institute, Canada)

Pr Philippe Ravaud (Paris Descartes University, INSERM, France),

*Authorization by CNIL (“Commission National de l’Information et des libertés”) whose remit is to protect participants’ personal data and the institutional review board of INSERM ethics committee (IRB xxxxxx) was obtained. In accordance with the law "Informatique et Libertés" of 6 January 1978 amended in 2004, you have the right to access and rectify any information concerning you, which can be exercised by contacting Professor Isabelle Boutron, Research Center Epidemiology and statistics Sorbonne Paris Cité (isabelle.boutron@htd.aphp.fr).*

*If you have any questions during your participation in this study, you can contact the person in charge of the study, Professor Isabelle Boutron, tel: +33 (0)1 42 34 78 33, mail:*
