# Supplementary material for: Accuracy in detecting inadequate research reporting by early career peer reviewers using an online CONSORT-based peer-review tool (COBPeer) versus the usual peer-review process: a cross-sectional diagnostic study
Source: BMC Med. 2019 Nov 19;17:205. doi: 10.1186/s12916-019-1436-0 (PMC6864983; doi:10.1186/s12916-019-1436-0)
Supplement: Supplementary file 9 — Additional file 9. General characteristics of reports of randomized controlled trials included in the study. Details of reports included in the study. [file 12916_2019_1436_MOESM9_ESM.docx]

Additional file 9. General characteristics of reports of randomized controlled trials included in the study.

| **Publishing journal** |  |
| --- | --- |
| *Annals of Emergency Medicine* | 16 (13.4) |
| *BMC Anesthesiology* | 13 (10.9) |
| *BMJ* | 12 (10.1) |
| *Trials* | 12 (10.1) |
| *BMC Musculoskeletal Disorders* | 9 (7.6) |
| *BMC Geriatrics* | 7 (5.9) |
| *BMJ Open* | 6 (5.0) |
| *Nutrition Journal* | 5 (4.2) |
| *BMC Public Health* | 5 (4.2) |
| *BMC Complementary and Alternative Medicine* | 4 (3.4) |
| *BMC Pediatrics* | 4 (3.4) |
| *BMC Gastroenterology* | 4 (3.4) |
| *BMC Medicine* | 4 (3.4) |
| *BMC Pulmonary Medicine* | 3 (2.5) |
| *BMC Cancer* | 3 (2.5) |
| *BMC Psychiatry* | 2 (1.7) |
| *BMC Urology* | 2 (1.7) |
| *BMC Infectious Diseases* | 2 (2.5) |
| *BMC Pregnancy and Childbirth* | 1 (0.8) |
| *BMC Ophtalmology* | 1 (0.8) |
| *BMC Neurology* | 1 (0.8) |
| *BMC Oral Health* | 1 (0.8) |
| *BMC Womens Health* | 1 (0.8) |
| *BMC Surgery* | 1 (0.8) |
| **Trial intervention** |  |
| *Drug* | 55 (46.2) |
| *Counselling* | 11 (9.2) |
| *Lifestyle* | 7 (5.9) |
| *Surgery* | 5 (4.2) |
| *Other* | 41 (34.5) |
| **Publication delay, days, median [IQR])^1^** | 192 [149;260] |
| **First peer-review delay, days, median [IQR]) ^1^** | 82 [55;118] |
| **Peer reviewers for the first round mean (SD) (min;max)** | 2.5 (1.0) (1;8) |
| **No. of centers involved** |  |
| **Multicentric** | 53 (44.5) |
| **Monocentric** | 53 (44.5) |
| **Unclear or not reported** | 13 (11.0) |
| **Participants randomized, median [Q1;Q3]** | 101 [54;224] |

Data are n (%) unless indicated.

^1^ missing data: 19
