# Supplementary material for: Accuracy in detecting inadequate research reporting by early career peer reviewers using an online CONSORT-based peer-review tool (COBPeer) versus the usual peer-review process: a cross-sectional diagnostic study
Source: BMC Med. 2019 Nov 19;17:205. doi: 10.1186/s12916-019-1436-0 (PMC6864983; doi:10.1186/s12916-019-1436-0)
Supplement: Supplementary file 10 — Additional file 10. Sensitivity analysis after exploring false-positive results. Analysis after reclassification of false-positive results by the reference standard. [file 12916_2019_1436_MOESM10_ESM.docx]

Additional file 10. Sensitivity analysis after exploring false-positive results.

|  | **Items 11a/11b (Blinding)** | | **Items 13a/13b (Participant flow)** | | **TOTAL**  **ALL CONSORT DOMAINS** | | **TOTAL**  **ALL**  **DOMAINS** | |
| --- | --- | --- | --- | --- | --- | --- | --- | --- |
|  | **ECR** | **Usual peer review** | **ECR** | **Usual peer review** | **ECR** | **Usual peer review** | **ECR** | **Usual peer review** |
| **Sensitivity (%)** | 57.7 | 19.2 | 93.5 | 21.7 | 86.0 | 20.1 | 83.9 | 19.3 |
| **Specificity (%)** | 77.6 | 83.6 | 23.3 | 80.8 | 61.7 | 77.5 | 63.7 | 80.1 |
| **Positive likelihood ratio** | 2.58 | 1.17 | 1.22 | 1.13 | 2.25 | 0.89 | 2.31 | 0.97 |
| **Negative likelihood ratio** | 0.55 | 0.97 | 0.28 | 0.97 | 0.23 | 1.03 | 0.25 | 1.01 |

ECR, early career peer reviewer
