# Supplementary material for: Accuracy in detecting inadequate research reporting by early career peer reviewers using an online CONSORT-based peer-review tool (COBPeer) versus the usual peer-review process: a cross-sectional diagnostic study
Source: BMC Med. 2019 Nov 19;17:205. doi: 10.1186/s12916-019-1436-0 (PMC6864983; doi:10.1186/s12916-019-1436-0)
Supplement: Supplementary file 8 — Additional file 8. Screenshot of the content of the standardized data extraction form used for the evaluation of the usual peer-review. Example of the data extraction form used. [file 12916_2019_1436_MOESM8_ESM.docx]

Additional file 8. Screenshot of the content of the standardized data extraction form used for the evaluation of the usual peer-review. The full online standardized data extraction form is available at https://forms.gle/ybykYbFanFu1nB8z9
